# Combination Therapy with Azithromycin and *Clostridium butyricum* Retains Anti-Diarrheal Efficacy but Partially Compromises Gut Microbiota Restoration Compared to Probiotics Monotherapy

**DOI:** 10.3390/microorganisms13122812

**Published:** 2025-12-10

**Authors:** Cai-Yun Wang, Meng-Yue Zhang, Hai-Ying Zhang, Yi-Meng Yang, Lu Zhang, Yi-Xuan Zhang

**Affiliations:** 1 School of Life Science and Biopharmaceutics, Shenyang Pharmaceutical University, Shenyang 110016, China; 2 Food Science and Engineering College, Beijing University of Agriculture, Beijing 102206, China; 3 Hangzhou Grand Biologic Pharmaceutical Inc., Hangzhou 100096, China

**Keywords:** *Clostridium butyricum*, azithromycin, infectious diarrhea, intestinal microbiota, metabolomics

## Abstract

Most probiotics require separate administration from antibiotics due to sensitivity issues. *Clostridium butyricum*, however, exhibits intrinsic resistance, making it a promising candidate for combined therapy against diarrhea. In this study, a diarrhea model was established in mice induced by *Escherichia coli*, followed by treatment with azithromycin (AZM), *C. butyricum* (RH_2_), or their combination (COM) to assess therapeutic efficacy. The results demonstrated that mice in RH_2_ and COM groups achieved full body weight recovery and significant alleviation of diarrhea, accompanied by normalized fecal *E. coli* loads, preserved tissue integrity, reduced pro-inflammatory cytokines (IL-1β, IL-6, TNF-α), and elevated anti-inflammatory IL-10. In contrast, AZM treatment led to sex-specific disparities in weight recovery and *E. coli* loads, and both sexes experienced relapse-prone diarrhea. Furthermore, the AZM group displayed shortened colons, sustained inflammatory infiltration, epithelial damage, and elevated IL-1β and male-specific IL-6. Gut microbiota analysis revealed that the COM group retained beneficial genera (e.g., *Parabacteroides*, *Blautia*) from the AZM group while uniquely enriching *Lachnospiraceae* taxa (e.g., *NK4A136_group*, *FCS020_group*). Untargeted metabolomics demonstrated the COM group activated GABA/arginine pathways, enhancing anti-inflammatory and barrier functions, whereas azithromycin disrupted butyrate synthesis and steroid metabolism. These findings highlight the advantage of combining *C. butyricum* with azithromycin for intestinal protection.

## 1. Introduction

Since the discovery of penicillin in 1928, antibiotics have been in use for nearly 100 years, saving countless lives. Antibiotics are effective and common drugs used in clinical practice to treat bacterial infections. The elimination of pathogenic bacteria also causes irreversible damage to beneficial bacteria, leading to a decrease in microbial diversity, proliferation of opportunistic pathogens, and impairment of metabolic functions [1,2,3]. Long-term antibiotic use disrupts the gut microbiota, resulting in side effects such as antibiotic-associated diarrhea (AAD) and indigestion. Probiotics, recognized as one of the most effective interventions, are widely used to regulate gut microbiota balance, enhance immune function, and improve metabolic health [4]. However, traditional probiotics, such as lactic acid bacteria and *Bifidobacterium*, are susceptible to the antibiotics commonly used in clinical practice (β-lactam, aminoglycoside, and certain macrolide) [5], so co-administration with antibiotics may impair their viability. Therefore, it is generally recommended to space probiotic and antibiotic dosing by 2–3 h if used concurrently [6]. This staggered dosing approach not only complicates the treatment regimen but also increases the risk of patient confusion of administration timing, which potentially prolongs the course of therapy and increases the risk of antibiotic resistance [7].

Currently, it has been reported that *Clostridium butyricum* exhibits widespread intrinsic or acquired resistance to aminoglycoside and β-lactam antibiotics compared to conventional probiotics. Its spore form also confers broad tolerance to harsh gastrointestinal conditions. The resistance to aminoglycoside antibiotics (e.g., gentamicin, kanamycin) is intrinsically linked to their highly conserved core genome architecture [8]. Due to their primary location in the chromosomal accessory regions, these resistance genes pose a low risk for horizontal transfer [9]. Resistance to β-lactam antibiotics (e.g., penicillins and cephalosporins) is directly linked to the carriage of β-lactamase genes. Identification of linear megaplasmids has revealed that most clinical isolates encode a β-lactamase on these large plasmids, which can hydrolyze the β-lactam ring and thereby inactivate the antibiotic [10]. In its spore form, *C. butyricum* exhibits significant tolerance to a wide range of antibiotics. When spores were exposed to ten clinically common antibiotics (including β-lactams, macrolides, aminoglycosides, and tetracyclines), their survival rate exceeded 89% in intestinal fluid and remained above 60% under gastric acid conditions. This tolerance is attributed to the physical barrier function of the spore against antibiotic penetration [11]. The spores enter a dormant state upon antibiotic exposure and resume growth upon dilution. Experimental studies further confirmed that even when co-administered with clindamycin (a lincosamide antibiotic), the intestinal colonization level of *C. butyricum* MIYAIRI 588 (CBM 588) showed no significant reduction compared to the monotherapy group [12,13]. The protective spore structure shields the bacterium from antibiotic-induced damage, providing a foundation for combined therapeutic use. In contrast, genera such as *Lactobacillus* and *Bifidobacterium* are prone to carrying transferable resistance genes (e.g., *tet*(W), *lnu*(A)), posing a risk of plasmid-mediated dissemination. These resistance genes often share homology with clinically significant resistance determinants (e.g., the *van*A gene in enterococci) [5,14]. Compared to these conventional probiotics, *C. butyricum* exhibits a more stable resistance profile and a lower risk of horizontal gene transfer, making it a promising candidate for development as a next-generation probiotic.

Beyond its favorable safety profile in terms of antibiotic resistance, the therapeutic potential of *C. butyricum* is primarily attributed to its direct functional contributions to gut health. As a key contributor to intestinal homeostasis, it competitively excludes opportunistic pathogens such as *E. coli* and *Salmonella* through niche occupation and nutrient competition, thereby promoting microbial ecological stability [15,16,17]. At the same time, it synthesizes vitamins and amino acids to facilitate the growth and colonization of beneficial bacteria, such as *Bifidobacterium* and *Lactobacillus*, which further enhance their resilience through cross-feeding interactions [18,19]. Furthermore, *C. butyricum* primarily produces short-chain fatty acids (SCFAs), such as butyrate, acetate, and small amounts of propionate in the animal intestine. Butyrate, as its core metabolic product, can promote cellular proliferation and repair, maintain mucosal integrity, and enhance barrier function [20,21]. Short-chain fatty acids can also lower the pH value of the intestinal tract, inhibit the proliferation of harmful bacteria such as *E. coli* and *Salmonella*, and create an appropriate growth environment for acidophilic probiotics such as *Bifidobacterium* and *Lactobacillus* [16,22]. Moreover, *C. butyricum* can stimulate the intestinal mucosal immune system, promote the proliferation and activation of immune cells such as macrophages and lymphocytes, and enhance their phagocytic ability and immune response [23,24]. At the same time, this bacterium can regulate the secretion of immune factors, promote the production of IL-10 and TGF-β, and inhibit the excessive secretion of pro-inflammatory factors (such as IL-6, TNF-α), thereby maintaining intestinal immune homeostasis and reducing inflammatory responses [15,20,25].

In this study, a mouse model of infectious diarrhea was successfully established through oral gavage of *E. coli*, with the primary objective of evaluating the potential interference of antibiotic co-administration on the therapeutic efficacy of *C. butyricum*. A comprehensive set of physiological and molecular parameters, including body weight changes, fecal consistency scores, fecal *E. coli* bacterial loads, colonic histomorphology, inflammatory cytokine levels, gut microbiota composition, and fecal metabolic profiles, was systematically monitored and compared following intervention with *C. butyricum* alone or in combination with azithromycin. The results provide critical experimental evidence and mechanistic insights that will inform the rational design of clinical regimens combining probiotics and antibiotics, ultimately contributing to improved therapeutic outcomes in the treatment of infectious diarrhea.

## 2. Materials and Methods

### 2.1. Cultivation and Preparation of Bacterial Strain

A single colony of strain *E. coli* SYP-B4131 (serotype O127:H6; EPEC, LEE^+^) was inoculated into 50 mL Luria-Bertani (LB) liquid medium and incubated at 37 °C at 200 rpm for 12 h. Then the culture was transferred into fresh LB medium with an inoculum ratio of 2:100 and continued to cultivate for 3 h. Afterwards, the bacterial strain was harvested by centrifugation at 8000× *g* for 10 min, and washed three times with phosphate-buffered saline (PBS) buffer. Finally, the bacterial pellets were resuspended in PBS buffer and diluted to approximately 1 × 10^10^ CFU/mL. The *Clostridium butyricum* RH_2_ was provided by Hangzhou Grand Biologic Pharmaceutical Inc. (Hangzhou, China), demonstrated high spore survival rates (>89%) in simulated intestinal fluid across 10 antibiotics from four major classes (β-lactams, macrolides, aminoglycosides, and tetracyclines) [11]. A suspension of 1 × 10^9^ CFU/mL was prepared by dissolving 0.5 g of a commercial spore powder (2.0 × 10^10^ CFU/g) in sterile PBS to a final volume of 10 mL.

### 2.2. Animals and Experimental Design

6~8-week-old BALB/c mice (half male and half female, 18–22 g) were obtained from Liaoning Changsheng Biotechnology Co., Ltd. (Shenyang, China). Laboratory Animal Use License No.: SYXK (Liao) 2021-0009. All animal procedures were conducted in strict compliance with the ethical standards established by the Laboratory Animal Management Committee of the Ministry of Health of the People’s Republic of China (Approval Code: SYPU-IACUC-S2024-1016-114). Laboratory animals were maintained under a 12 h light/dark cycle in well-ventilated conditions at 24 ± 2 °C with 50–70% humidity. They were acclimatized for a week in individual cages with ad libitum access to sterile food and water.

50 BLAB/c mice were randomly assigned to five groups (*n* = 10 per group, with five males and five females): control (NC), model (MOD), azithromycin (AZM), *C. butyricum* (RH_2_), and azithromycin and *C. butyricum* combination therapy group (COM). During the modeling phase, mice in the NC group received daily oral gavage with 0.2 mL sterile PBS, whereas all other mice were administered 0.2 mL of *E. coli* SYP-B4131 bacterial suspension at approximately 1 × 10^10^ CFU/mL for three consecutive days.

On the second day of the modeling period, a 15-day therapeutic intervention was initiated. Mice in the NC and MOD groups received daily oral gavage of 0.3 mL sterile PBS. The AZM group was administered 65 mg/kg azithromycin plus 0.2 mL sterile PBS daily, whereas the RH_2_ group received 0.2 mL of a fresh *C. butyricum* suspension (1 × 10^9^ CFU/mL, a dose of 2 × 10^8^ CFU) [26,27] with 0.1 mL sterile PBS. The COM group was treated with a combination of 0.2 mL fresh *C. butyricum* suspension (1 × 10^9^ CFU/mL) and 65 mg/kg azithromycin solution via daily intragastric gavage.

### 2.3. Sample Collection and Processing

Serum collection: Following a 15-day treatment period and a 12-h fasting period (with free access to water), mice were anesthetized by an intraperitoneal injection of a ketamine (100 mg/kg) and xylazine (10 mg/kg) cocktail. Blood sampling via retro-orbital puncture using glass capillaries commenced only after the absence of a pedal withdrawal reflex was confirmed, ensuring the animals did not experience pain or distress. Whole blood samples were maintained in sterile 1.5 mL Eppendorf tubes for 60 min, followed by centrifugation at 3500× *g* for 10 min. The resulting serum supernatant was carefully aliquoted for subsequent analysis.

Collection of feces samples: The mouse was gently restrained with the left hand while the right index finger performed a clockwise circular massage along the abdominal midline to stimulate defecation. Fresh fecal pellets were collected directly into sterile Eppendorf tubes for subsequent *E. coli* quantification. For other analyses, fecal samples were obtained by housing mice individually in sterile, bedding-free cages to allow for natural excretion.

Organ and luminal content collection: At the end of the treatment, mice were euthanized by cervical dislocation. The colorectum was carefully excised, flushed with sterile 0.85% NaCl to remove luminal contents. One quarter was fixed in 4% paraformaldehyde (PFA) for histology, and the remainder was snap-frozen in liquid nitrogen and stored at −80 °C for subsequent analysis. The cecal contents were aseptically collected into sterile Eppendorf tubes following dissection.

**Figure 1:**
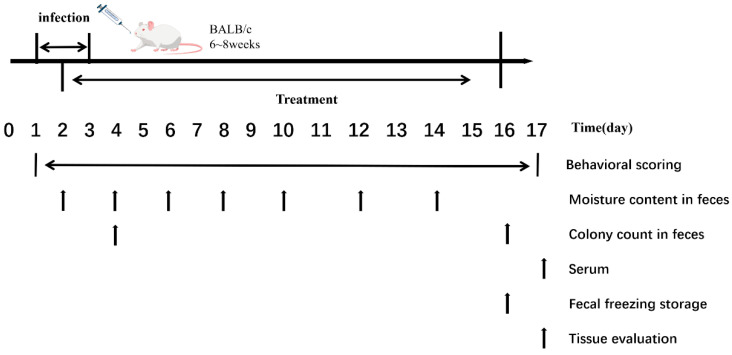
Experimental Procedures and Sampling Time Points.

The experimental procedures and sampling time points are shown in Figure 1.

### 2.4. Measurement of Body Weight and Fecal Moisture Content

Body weight was recorded daily for all mice. Concurrently, general activity and mental state were monitored, with observations focused on signs such as reduced spontaneous activity, a hunched posture, lethargic behavior, and a coat that was either piloerected or dull and ruffled. Behavioral and mental status were assessed every two days using the scoring criteria provided in Table 1.

Fecal samples were collected at fixed intervals every 48 h and assessed for consistency using the scoring criteria detailed in Table 2. The wet weight of fecal samples was measured immediately after collection, followed by oven drying at 70 °C for 20 h to determine the dry weight. The fecal moisture content was calculated as follows:

Moisture Content (%) = [(Wet Weight − Dry Weight)/Wet Weight] × 100%

### 2.5. Determination of E. coli Counts in Feces

Fecal samples were collected from all experimental groups at pre-modeling, post-modeling, and post-treatment stages, immediately transferred to sterile 1.5 mL tubes, and weighed. Each sample was homogenized in 1 mL of sterile PBS by vigorous vortexing. After serial 10-fold dilutions, 100 μL aliquots from appropriate dilutions were plated on MacConkey agar and incubated at 37 °C for 24 h. Pink colonies (indicating lactose-fermenting *E. coli*) were counted, and the results were expressed as colony-forming units per gram of fecal sample (CFU/g).

### 2.6. Observation of Colorectal Tissue Morphology

Following euthanasia, the entire colorectum was carefully excised, and its length was measured using a sterile ruler. Histological processing, including paraffin sectioning, H&E staining, and slide scanning, was performed by ServiceBio Technology Co., Ltd. (Wuhan, China). Colon tissue pathology was evaluated according to established scoring criteria [28], with three random non-continuous fields captured per sample at 200× magnification for microscopic assessment.

### 2.7. Assessment of Immune Factors

Serum levels of TNF-α, IL-1β, IL-6, and IL-10 were quantified using commercial ELISA kits (TNF-α, Cat. No. KS10484; IL-1β, Cat. No. KS11646; IL-6, Cat. No. KS18212; IL-10, Cat. No. KS10138; Shanghai Keshun Biological Technology Co., Ltd., Shanghai, China). Samples were appropriately diluted to ensure OD values were within the linear range of the standard curve, and absorbance was measured at 450 nm.

### 2.8. Microbial Composition Analysis of Gut Microbiota

Fecal samples were randomly selected from two male and two female mice for microbial composition analysis of gut microbiota. Total microbial genomic DNA was then extracted from these samples using the Mag-Bind Soil DNA Kit (Omega, Norcross, GA, USA). The V4 region of the 16S rRNA gene was amplified with barcoded primers under the following conditions: 98 °C for 1 min; 30 cycles of 98 °C/10 s, 50 °C/30 s, 72 °C/30 s; final extension at 72 °C for 10 min. PCR products (400–450 bp) were purified using QIAquick Gel Extraction Kit (Qiagen GmbH, Hilden, Germany). Library preparation was conducted using the TruSeq^®^ DNA PCR-Free Sample Preparation Kit (Illumina, Inc., San Diego, CA, USA). Following successful quality assessment via Qubit quantification and library validation, the libraries were sequenced on an Illumina NovaSeq 6000 instrument (Illumina, Inc., San Diego, CA, USA) with a PE250 read length configuration.

Alpha Diversity Analysis: Alpha diversity was assessed to evaluate species composition variation across samples. Clean reads from all samples were processed in QIIME 2 for clustering and taxonomic annotation using the Silva 138 database (https://www.arb-silva.de/, accessed on 28 November 2024) for 16S/18S rRNA gene annotation and the UNITE database (https://unite.ut.ee/, accessed on 28 November 2024) for ITS region annotation. All samples were rarefied to the minimum sequence count to ensure even sequencing depth. Species richness was measured by the Chao1 indices, and community diversity was quantified using the Shannon indices. Rarefaction curves were generated for each index to verify sequencing depth adequacy and microbial community profiling saturation.

Beta Diversity Analysis: To visualize the abundance profiles of each sample across different taxonomic levels, Krona (version 2.8.1) (https://github.com/marbl/Krona/wiki, accessed on 28 November 2024) was used to generate interactive visualizations based on species annotation results and abundance data per sample. Venn diagrams, bar plots, and heatmaps were used to compare species composition across samples and groups. Similarity and dissimilarity in community structure were further examined using dimensionality reduction methods, including Principal Coordinates Analysis (PCoA), Principal Component Analysis (PCA), and Non-Metric Multidimensional Scaling (NMDS). Statistical significance of group differences in microbial composition was assessed using ANOSIM and PERMANOVA (Adonis) tests.

STAMP was employed to identify statistically significant differences in taxonomic abundance across sample groups. LEfSe analysis was conducted to determine microbial biomarkers exhibiting differential abundance among experimental conditions.

### 2.9. Metabolite Analysis of Feces

Samples were randomly selected from two male and two female mice, and thawed at 4 °C, and mixed with pre-chilled methanol/acetonitrile/water (2:2:1, *v*/*v*). After vortexing, the mixtures were sonicated in an ice-water bath for 30 min and subsequently incubated at −20 °C for 10 min, and then centrifuged at 14,000× *g*, 4 °C for 20 min. The resulting supernatant was vacuum-dried and reconstituted in 100 μL of acetonitrile/water (1:1, *v*/*v*) followed by vigorous vortexing for 1 min. After a final centrifugation at 14,000× *g*, 4 °C for 15 min, the clear supernatant was taken for subsequent analysis.

Samples were separated on an Agilent 1290 Infinity LC ultra-high performance liquid chromatography (UHPLC) system equipped with a HILIC column (ACQUITY UPLC BEH Amide 1.7 μm, 2.1 mm × 100 mm Column, Waters Corporation, Milford, MA, USA) (25 °C, 0.5 mL min^−1^, 2 µL injection). The chromatographic method employed a binary mobile phase system with water containing 25 mM ammonium acetate and 25 mM ammonia (Mobile Phase A) and acetonitrile (Mobile Phase B), coupled with a multi-step gradient profile. The gradient elution program was set as follows: an isocratic elution with 95% B was maintained from 0 to 0.5 min; followed by a linear gradient from 95% to 65% B over 0.5 to 7 min; then a further linear decrease to 40% B from 7 to 8 min; subsequently, an isocratic elution at 40% B was held from 8 to 9 min; thereafter, a rapid linear increase to 95% B was performed from 9 to 9.1 min; and finally, 95% B was maintained from 9.1 to 12 min for column re-equilibration. Samples were kept at 4 °C in the autosampler and analyzed in random order to minimize signal drift; QC samples were interspersed to monitor system stability.

Following chromatographic separation, the samples were analyzed by mass spectrometry on an AB SCIEX Triple TOF 6600 system (SCIEX, Framingham, MA, USA). Data acquisition was carried out in both positive and negative electrospray ionization (ESI) modes.

### 2.10. Statistical Analysis

All data were tested for normality (Shapiro-Wilk test) and homogeneity of variances (Levene’s test). Parametric data were analyzed by one-way ANOVA with Tukey’s test, while non-parametric data were analyzed by the Kruskal-Wallis test with Dunn’s test or PERMANOVA. *p* values of <0.05 were considered statistically significant. Multiple comparisons were carried out using Tukey’s Honestly Significant Difference (HSD) test. The Benjamini-Hochberg FDR procedure controlled for multiple comparisons in high-throughput analyses.

## 3. Results

### 3.1. Changes in Diarrhea Symptoms

**Figure 2:**
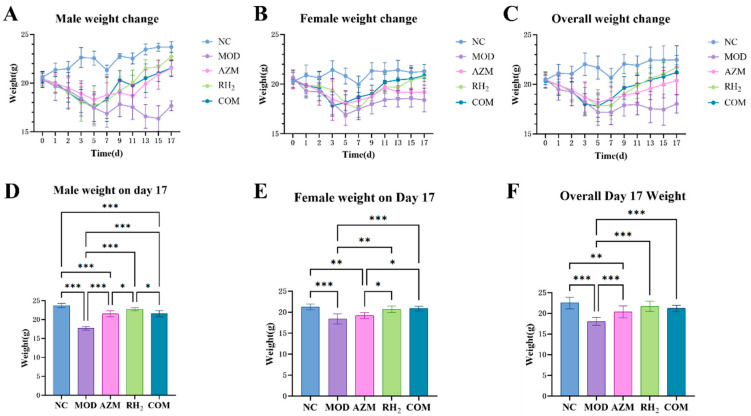
Body weight changes in mice. (**A**) Body weight changes in male mice; (**B**) Body weight changes in female mice; (**C**) Overall body weight changes; (**D**) Body weight of male mice on day 17; (**E**) Body weight of female mice on day 17; (**F**) Overall body weight on day 17. The data are presented as the mean ± standard deviation (SD). (* *p* < 0.05, ** *p* < 0.01, *** *p* < 0.001, For males and females, *n* = 5 respectively; for the overall group, *n* = 10).

Three days post-infection, a progressive decline in body weight was observed, reaching its nadir on days 5–7, followed by gradual recovery. As shown in Figure 2A,B, male mice exhibited more pronounced weight fluctuations than females. The MOD group showed a fluctuating decline, with the lowest weight on day 15 (19.73% reduction from baseline), ultimately stabilizing at a 13.25% decrease. In contrast, the RH_2_, AZM, and COM groups reached their lowest weight on day 5 and then began to recover. The RH_2_ group recovered rapidly, with a final weight of 11.70% above initial weight, while the AZM and COM groups recovered more slowly, finishing at 5.08% and 6.42% above baseline, respectively (Figure 2A,D).

While female mice showed relatively smaller weight fluctuations. The MOD group reached the lowest weight on day 5 (18.10% decrease), gradually recovering to a final 10.94% reduction (Figure 2B). The RH_2_ group began rapid recovery after day 7, ultimately achieving a 0.68% increase relative to initial weight. The COM group showed no significant difference from the RH_2_ group at the endpoint, with a final weight of 2.25% above baseline (Figure 2E). Notably, the AZM group initially recovered well but slowed later, ultimately resulting in a 6.25% decrease from the initial body weight.

Overall, the MOD group exhibited the slowest weight recovery, followed by the AZM group, while the RH_2_ group showed the most favorable recovery (Figure 2C). Post-treatment, the COM group demonstrated no significant difference in body weight compared to the RH_2_ group (Figure 2F).

Additionally, after model establishment, the MOD group exhibited hunched posture, reduced activity and lethargy, huddling and shivering, and dull fur with significant hair loss, which were milder in other groups (Figure S1). The AZM group exhibited fluctuating behavioral and mental states, while the RH_2_ and COM groups maintained stable recovery.

### 3.2. Changes in Fecal Water Content

**Figure 3:**
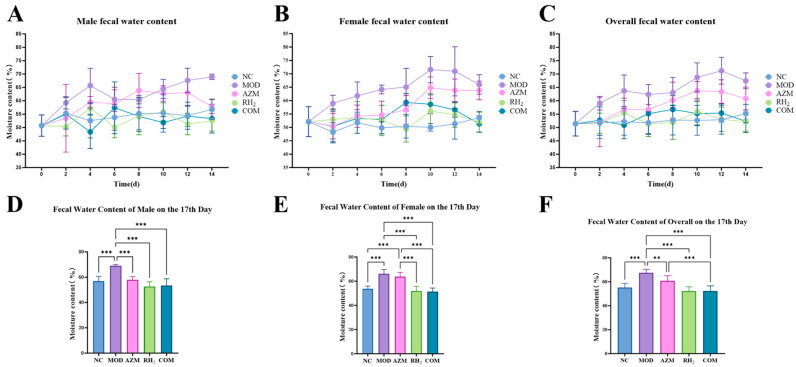
Changes in fecal water content of mice. (**A**) Male fecal moisture content; (**B**) Female fecal moisture content; (**C**) Overall fecal moisture content; (**D**) Male fecal moisture content on day 17; (**E**) Female fecal moisture content on day 17; (**F**) Overall fecal moisture content on day 17. The data are presented as the mean ± standard deviation (SD). (** *p* < 0.01, *** *p* < 0.001, For males and females, *n* = 5 respectively; for the overall group, *n* = 10).

Fecal consistency scores and water content analysis (Figure 3 and Figure S2) indicated persistent diarrhea in the MOD group, with maximum scores of 4 and fecal water content of 67.49% (males) and 66.00% (females). The AZM group exhibited recurrent diarrhea, with scores fluctuating between 2–3 points and water content of 57.85% (males) and 63.79% (females). In contrast, the RH_2_ and COM groups showed significant improvement, with scores reduced to 0–1 by the endpoint and water content of 52.49% (male) and 51.90% (female) in RH_2_, and 53.33% (male) and 51.34% (female) in COM. Although the COM group initially recovered more slowly, its ultimate fecal water content at the study endpoint was comparable to that of the RH_2_ group, with no significant difference detected.

### 3.3. Changes in E. coli Counts

**Figure 4:**
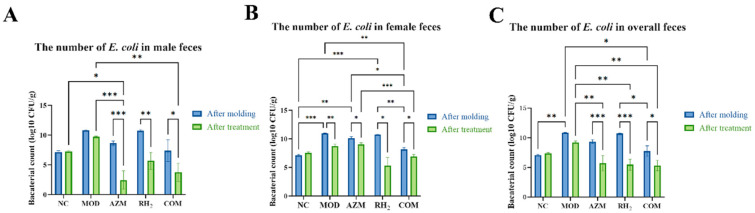
Fecal *E. coli* counts in mice. (**A**) Male; (**B**) Female; (**C**) Overall mean. The data are presented as the mean ± standard deviation (SD). (* *p* < 0.05, ** *p* < 0.01, *** *p* < 0.001, For males and females, *n* = 5 respectively; for the overall group, *n* = 10).

After modeling, the MOD group showed a significant increase in fecal *E. coli* counts. Post-treatment, all three treatments (AZM, RH_2_, and COM) significantly reduced *E. coli* load compared to the MOD group. Although the efficacy of the treatments exhibited sex-specific variation, with the most pronounced reduction observed in AZM-treated male mice, the interventions were uniformly effective in suppressing *E. coli*, as final bacterial loads dropped to sub-normal levels across all groups (Figure 4).

### 3.4. Analysis of Rectal Pathological Tissues

**Figure 5:**
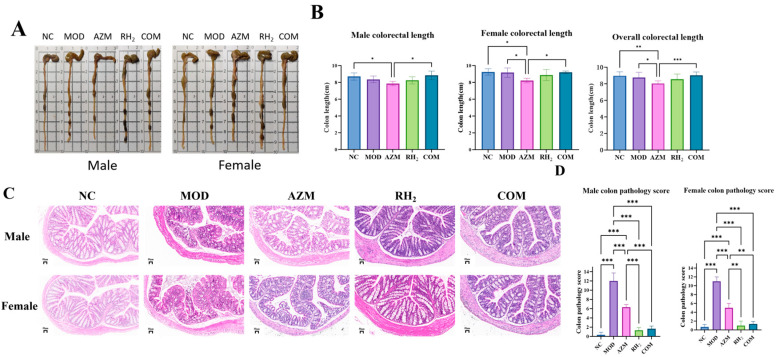
Evaluation of colorectal tissue in mice after treatment. (**A**) Colorectum length of Mouse; (**B**) Mean colorectal length. The data are presented as the mean ± standard deviation (SD). (* *p* < 0.05, ** *p* < 0.01, *** *p* < 0.001, For males and females, *n* = 5 respectively; for the overall group, *n* = 10); (**C**) Histology of colon tissue (H&E staining, ×200; Scale bars: 50 μm); (**D**) Colon pathology score. The data are presented as the mean ± standard deviation (SD). (** *p* < 0.01, *** *p* < 0.001, For males and females, *n* = 5 respectively; for the overall group, *n* = 10).

To evaluate the impact of *C. butyricum* on colon tissue, colorectal length and histology were assessed to examine mucosal integrity, inflammatory infiltration, and epithelial damage. Measurement of colorectal length showed that long-term azithromycin administration significantly shortened the colorectum, while supplementation with *C. butyricum* in the COM group effectively mitigated this effect (Figure 5A,B).

Histological analysis revealed that the NC group exhibited intact architecture with densely arranged crypts and abundant goblet cells in the lamina propria. In contrast, the MOD group showed severe disruption: disorganized and irregular crypts, focal erosions, extensive epithelial sloughing, crypt loss replaced by connective tissue, and focal inflammatory aggregates, which confirmed successful establishment of the model. The AZM group displayed inflammatory cell infiltration in the lamina propria with focal organization, along with extensive epithelial detachment and erosion (Figure 5C).

In comparison, both the RH_2_ and COM groups showed minimal structural damage and mild inflammatory infiltration. As shown in Figure 5D, all treatment groups exhibited significant recovery in colonic injury compared to the MOD group. Notably, *C. butyricum* intervention in the COM group enhanced epithelial integrity and goblet cell numbers while reducing inflammatory areas relative to the AZM group, highlighting the therapeutic efficacy of the probiotic.

### 3.5. Measurement of Inflammatory Factor

**Figure 6:**
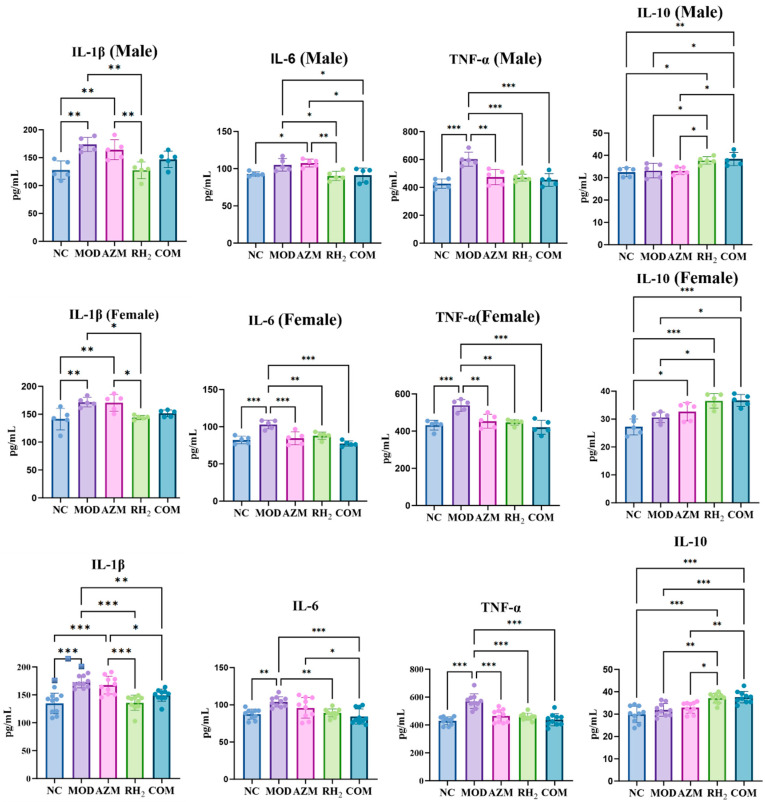
Serum cytokine levels. (* *p* < 0.05, ** *p* < 0.01, *** *p* < 0.001, For males and females, *n* = 5 respectively; for the overall group, *n* = 10).

To evaluate the effects of *C. butyricum* on cytokine regulation, serum levels of pro- and anti-inflammatory cytokines were measured. After treatment, pro-inflammatory cytokines (IL-1β, IL-6, TNF-α) remained elevated in the MOD group, indicating inflammation still persisted in the mice (Figure 6). The AZM group showed partial and sex-dependent cytokine suppression. Specifically, IL-1β in females and IL-6 in males showed no significant changes relative to the MOD group, whereas IL-1β in males, IL-6 in females, and TNF-α were downregulated to varying degrees. In contrast, the COM groups achieved near-complete normalization of pro-inflammatory cytokine levels and a moderate increase in IL-10, which was consistent with the RH_2_ group. These findings indicate that azithromycin did not compromise the immunomodulatory benefits of *C. butyricum* in the combination therapy, underscoring the compatibility of this combinatorial approach.

### 3.6. Analysis of Microbial Community Structure

**Figure 7:**
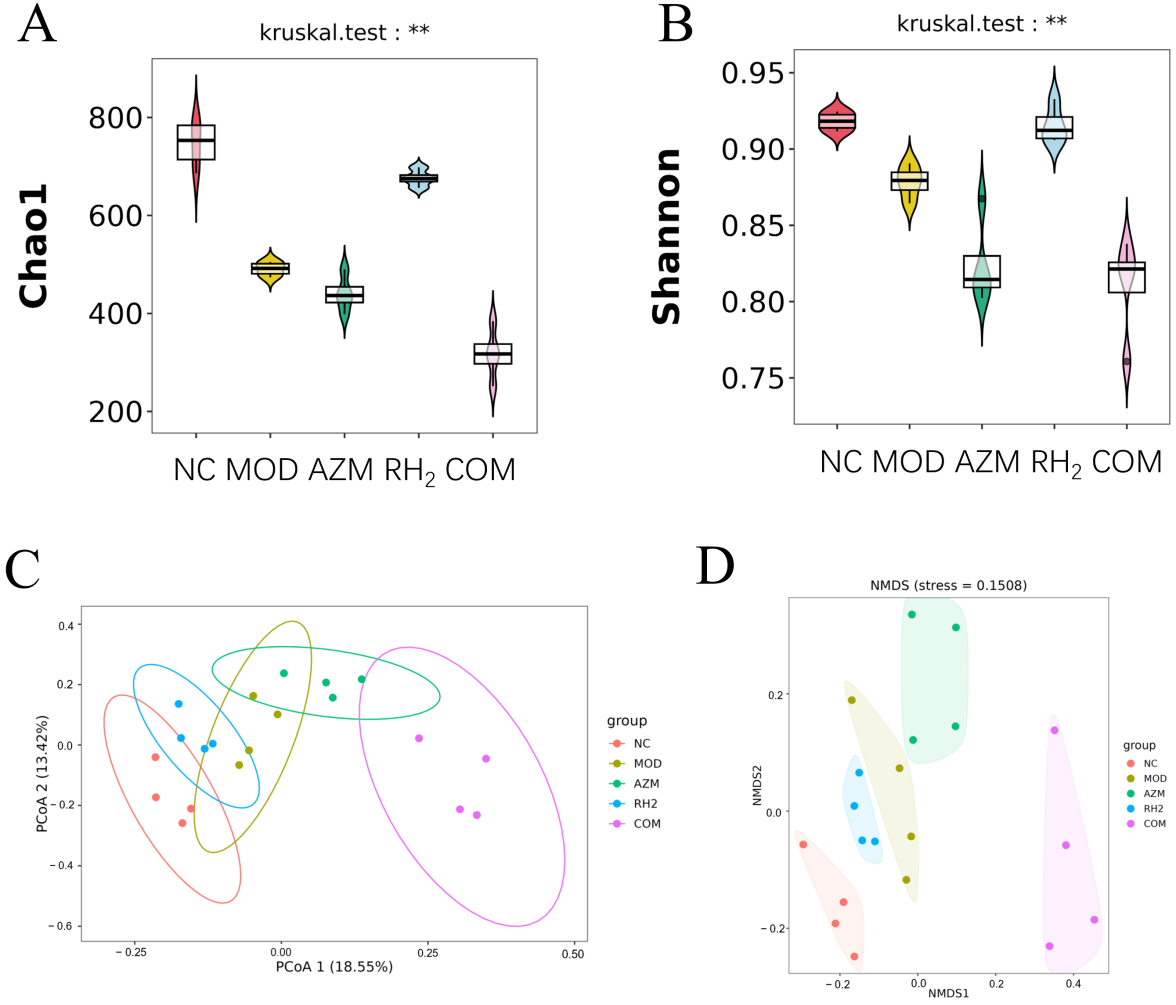
Microbial diversity analysis. (**A**) Chao 1 index; (**B**) Shannon index; (**C**) PCoA; (**D**) NMDS (** *p* < 0.01, For males and females, *n* = 2 respectively; for the overall group, *n* = 4).

Alpha and Beta diversity Analysis: To evaluate the effect of *C. butyricumin* on the gut microbiota of *E. coli*-induced diarrhea, high-throughput sequencing was performed to assess microbial diversity. As shown in Figure 7A,B, the Chao1 index and Shannon indices were significantly decreased in the MOD, AZM, and COM groups compared to the NC group (*p* < 0.05, *n* = 4), indicating that both diarrheal infection and long-term antibiotics use disrupted the microbial diversity. In contrast, the RH_2_ group exhibited no significant difference from the NC group (*p* > 0.05, *n* = 4), suggesting that *C. butyricum* intervention can effectively restore the alpha diversity to a level similar to healthy controls. In summary, *E. coli*-induced diarrhea led to gut microbiota dysbiosis and reduced alpha diversity. Treatment with *C. butyricum* successfully reversed these changes, whereas prolonged antibiotic use decreased species richness and evenness, an effect that was partially mitigated by concurrent *C. butyricum* supplementation.

A β-diversity analysis was performed to compare microbial community structure across groups, revealing compositional shifts under different experimental conditions. The Principal Coordinates Analysis (PCoA) plot, based on Bray-Curtis distances, shows clear separation among groups along the first two principal coordinates. The NC and RH_2_ groups clustered closely in the left section of the plot, indicating that the microbial community in the RH_2_ group shifted towards a state resembling that of the healthy controls. In contrast, the MOD and AZM groups formed a separate cluster in the upper-right section, demonstrating that both infection and antibiotic treatment significantly disrupted the gut microbiota. Notably, the COM group shifted away from the AZM cluster, suggesting that co-administration of *C. butyricum* partially counteracted the dysbiotic effects of azithromycin (Figure 7C).

The Non-metric Multidimensional Scaling (NMDS) analysis yielded a consistent grouping pattern with the PCoA results. The spatial distribution of samples in the NMDS plot confirms the same ecological relationships: NC and RH_2_ groups showed high similarity, MOD and AZM groups formed a distinct dysbiotic cluster, and the COM group exhibited a transitional state toward a healthier microbiota (Figure 7D). The beta diversity analyses consistently demonstrate that *C. butyricum* intervention effectively promotes a shift towards a healthy gut microbiota structure in mice with infectious diarrhea. While azithromycin treatment resulted in significant dysbiosis similar to the model group, the concurrent administration of *C. butyricum* in the COM group partially mitigated these adverse effects, supporting the potential of probiotic-antibiotic combination therapy in managing antibiotic-associated microbial imbalances.

**Figure 8:**
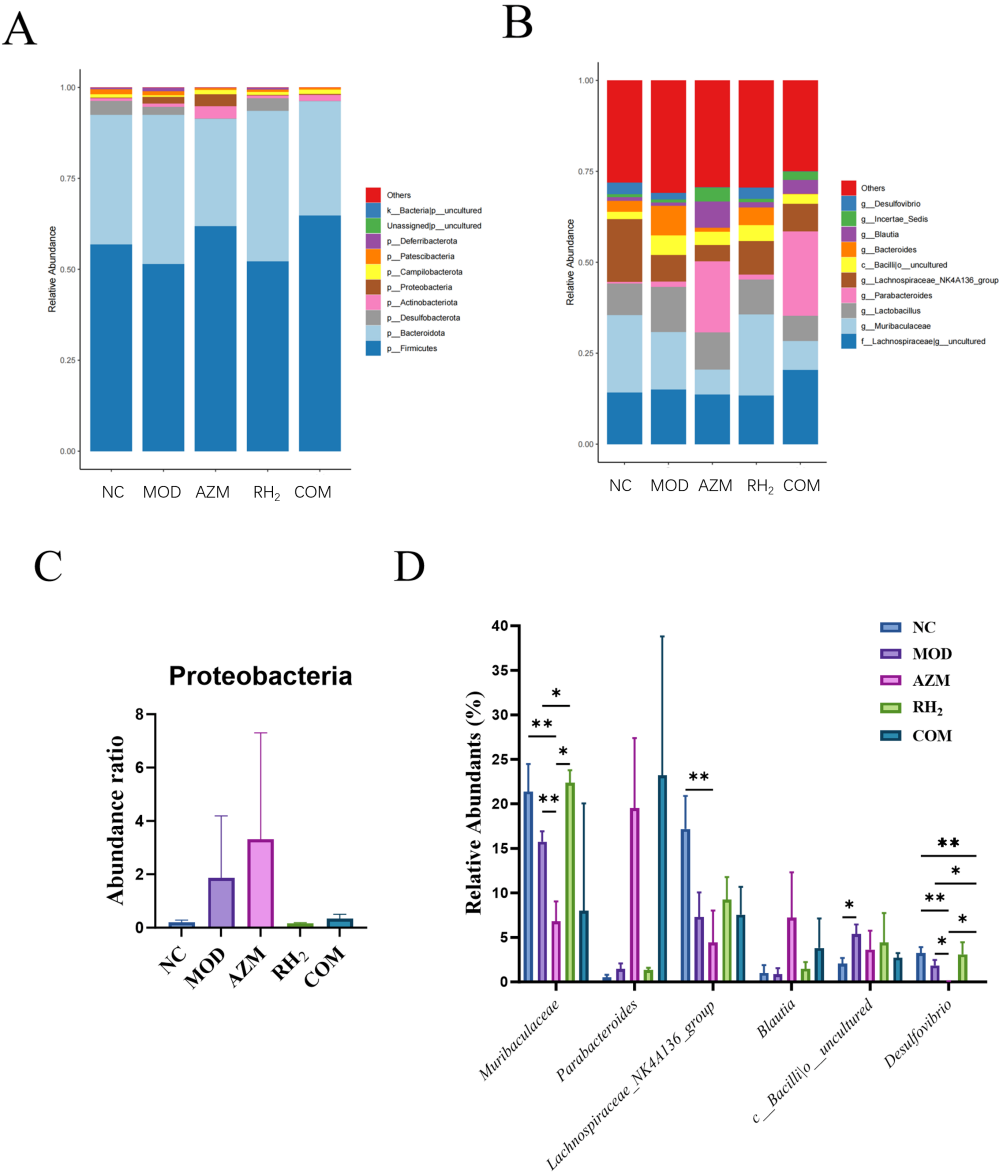
Changes in abundance of mouse gut microbiota. (**A**) Phylum-level relative abundance; (**B**) Genus-level relative abundance; (**C**) Proteobacteria relative abundance; (**D**) Abundance of specific genera. (* *p* < 0.05, ** *p* < 0.01).

Species analysis of the intestinal microbiota: Based on taxonomic annotation, the top 10 most abundant species per group were selected to generate stacked bar charts of relative species abundance. At the phylum level, the eight most abundant taxa across all groups were p_Firmicutes, p_Bacteroidota, p_Desulfobacterota, p_Actinobacteriota, p_Proteobacteria, p_Campilobacterota, p_Patescibacteria, and p_Deferribacterota. p_Firmicutes, p_Bacteroidota, and p_Desulfobacterota constituted the dominant phyla, collectively accounting for over 90% of total abundance (Figure 8). The MOD and AZM groups showed increased relative abundance of p_Proteobacteria, a phylum containing numerous opportunistic pathogens, suggesting that *E. coli* colonization disrupted gut microbial structure and promoted expansion of harmful bacteria. Similarly, prolonged azithromycin use induced dysbiosis and favored pathogenic proliferation. In contrast, the COM group exhibited no increase in p_Proteobacteria, indicating that *C. butyricum* intervention effectively suppressed pathogenic growth. Compared to other groups, the AZM and COM groups had higher abundance of p_Actinobacteriota but lower proportions of p_Desulfobacterota and p_Deferribacterota. The MOD group showed reduced p_Desulfobacterota and elevated p_Deferribacterota relative to the NC group. Overall, the RH_2_ group displayed a microbial composition closest to the NC group, underscoring the efficacy of *C. butyricum* in restoring gut microbiota balance.

At the genus level, the dominant bacterial taxa included g__uncultured_f__*Lachnospiraceae*, g_*Muribaculaceae*, g_*Lactobacillus*, g_*Parabacteroides*, and g_*Lachnospiraceae_NK4A136_group* (Figure 8). Compared to the MOD group, the RH_2_ group showed a significant increase in the abundance of g_*Muribaculaceae* (*p* < 0.05, *n* = 4), reaching levels comparable to the NC group, while the AZM (*p* < 0.01, *n* = 4) and COM groups exhibited reductions. g_*Muribaculaceae*, a family within the Bacteroidota, ferments complex polysaccharides to produce acetate and propionate, providing energy to the host [29]. It also supports gut barrier function and competitively inhibits pathogens, contributing to microecological balance [30]. The RH_2_ and COM groups showed increased abundance of g_*Lachnospiraceae_NK4A136_group* compared to the MOD group, whereas the AZM group showed a decrease. *Lachnospiraceae* produce butyrate, which is critical for maintaining barrier integrity, anti-inflammatory responses, and immune regulation [31]. They also inhibit pathogens such as *E. coli* and *Salmonella* through niche competition and nutrient exclusion. In addition, g_*Bacteroides* abundance was reduced in the RH_2_, AZM, and COM groups relative to the MOD group. Notably, the AZM and COM groups displayed higher abundances of g_*Parabacteroides* and g_*Blautia*, along with reduced levels of uncultured *Bacilli* (c_*Bacilli*_uncultured) and g_*Desulfovibrio* (*p* < 0.05, *n* = 4). Both g_*Blautia* and g_*Parabacteroides* have been reported to inhibit pro-inflammatory cytokines and maintain intestinal immune homeostasis [32,33], whereas low g_*Desulfovibrio* levels help mitigate inflammation, modulate immunity, and protect barrier function [34].

Overall, treatment with *C. butyricum* promoted the proliferation of beneficial bacteria in mice with *E. coli*-induced infectious diarrhea, resulting in a microbial composition similar to that of healthy mice. Although long-term antibiotic use significantly altered microbiota abundance, co-administration of *C. butyricum* positively influenced the structure of dominant intestinal flora.

Analysis of Gut Microbiota Species Differences: To further identify microbial taxa contributing to community differences, LEfSe (Linear Discriminant Analysis Effect Size) was employed to analyze significantly differential microbial biomarkers across comparison groups. As shown in Figure S3, the MOD group was characterized by microbial biomarkers predominantly associated with pro-inflammatory functions, including genera such as g_*Alistipes*, g_*Mucispirillum*, and g_*Gemella*. The AZM group exhibited significant gut microbiota dysbiosis, marked by substantial enrichment of opportunistic pathogens such as g_*Enterorhabdus*, g_*Escherichia-Shigella*, and g_*Erysipelatoclostridium*. The co-occurrence of facultative anaerobes (e.g., g_*Enterococcus*, g_*Streptococcus*) and strict anaerobes (e.g., g_*Anaerorhabdus*, g_*Anaerosporomusa*) suggests potential intestinal hypoxia or mucosal injury. In contrast, the RH_2_ group exhibited a significant enrichment of butyrate-producing bacteria, including g_*Roseburia*, g_*Lachnospiraceae_UCG-001*, and g_*Ruminococcaceae*, which, together with g_*Muribaculaceae*, established a symbiotic microbial network primarily composed of short-chain fatty acid-producing and dietary fiber-degrading taxa. Following *C. butyricum* intervention, the COM group exhibited a markedly shifted biomarker profile. The community was dominated by probiotics conferring metabolic and barrier-protective functions, such as g_*Parabacteroides* and g_*Lachnospiraceae_FCS020_group*. The detection of genera such as g_*Staphylococcus* and g_*Erysipelatoclostridium* is noted; however, considering the community dominated by beneficial bacteria and a marked antidiarrheal effect, their presence likely reflects a constrained ecological niche within a therapeutically restructured and functionally resilient gut microbiome.

### 3.7. Fecal Metabolomic Analysis in Mice

To further compare the effects of antibiotics on the metabolic function of *C. butyricum*, untargeted metabolomics was performed on mouse fecal samples. A total of 66, 38, and 77 differential metabolites were identified in the AZM, RH_2_, and COM groups, respectively (Figure S4). Comparative analysis revealed that the COM group shared 15 differential metabolites with the RH_2_ group and 33 with the AZM group (Figure S5). Eight metabolites were common to all three groups, namely 1-deoxy-D-xylulose 5-phosphate, cholesteryl sulfate, DL-normetanephrine, hydrocortisone, indoxyl sulfate, phenyllactic acid, sarcosine, and taurochenodeoxycholate (Figure S6), suggesting their potential association with the pathogenesis of infectious diarrhea. Butyrate, a primary metabolite of *C. butyricum*, is fundamental to gut health, influencing epithelial barrier function, immune regulation, and energy metabolism. As a key energy source for colonic epithelial cells, fecal butyrate levels reflect a dynamic balance between microbial production and host utilization, which may result in a non-significant net change in fecal concentration. Given the central role of butyrate as the primary functional metabolite of *C. butyricum*, its levels were analyzed as a pre-specified target. The relative abundance of butyric acid and shared differential metabolites is shown in Figure S6.

Metabolic pathway enrichment analysis of differential metabolites from the AZM, RH_2_, and COM groups was performed using MetaboAnalyst 6.0 to compare alterations in key metabolic pathways across therapeutic interventions. Pathways with *p* < 0.05 and the highest enrichment ratios were prioritized for analysis. Results revealed that the AZM group was primarily associated with steroid hormone biosynthesis and butanoate metabolism. The RH_2_ group exhibited significant enrichment in vitamin B6 metabolism and lysine degradation. In contrast, the COM group showed concurrent enrichment in butanoate metabolism, vitamin B6 metabolism, and arginine and proline metabolism (Figure S7), reflecting a broader metabolic restoration profile.

**Figure 9:**
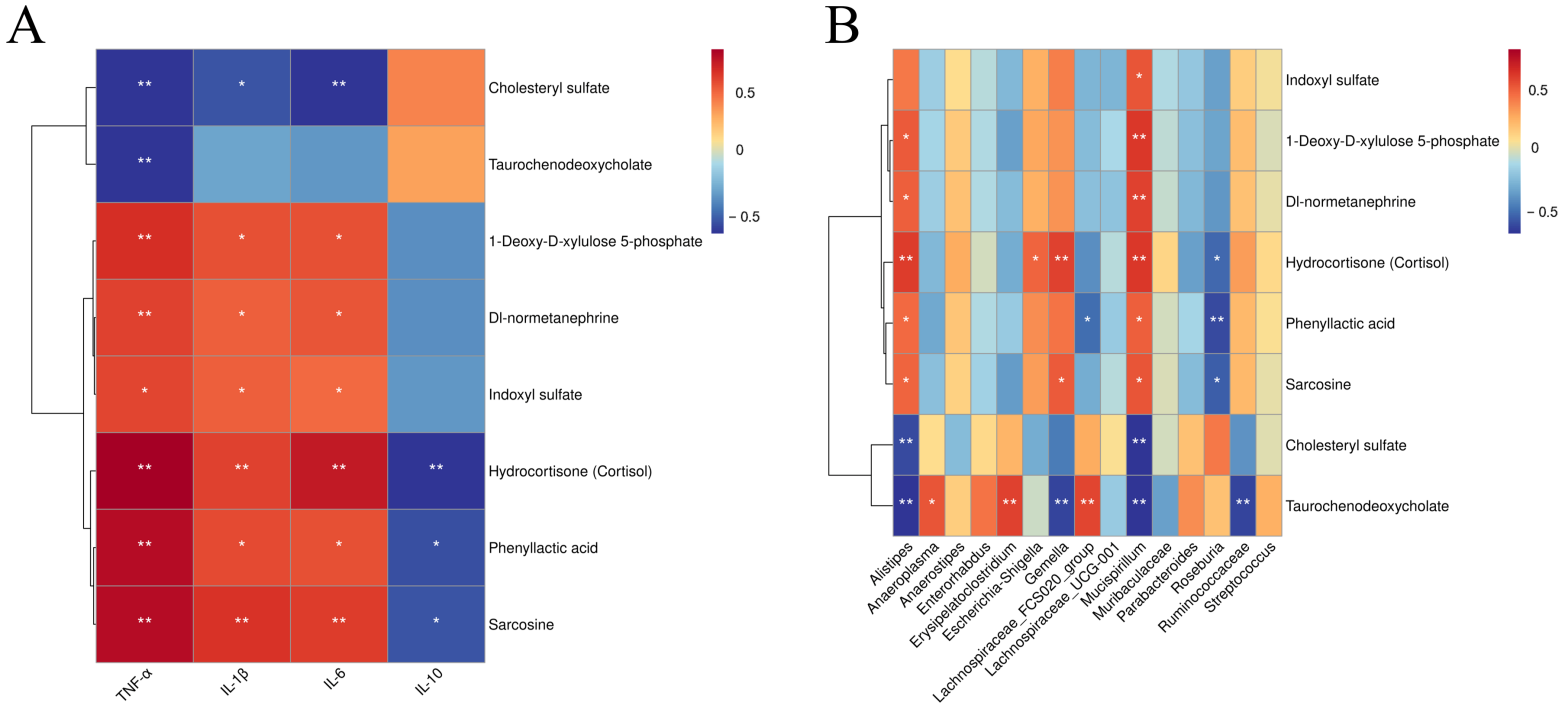
Heatmap of fecal metabolite correlations with serum cytokines and signature microbiota. (**A**) Correlation between metabolites and cytokines; (**B**) Correlation between metabolites and microbiota. Strength (Spearman’s ρ value) and significance of correlations are shown as colors in shades (red, positive correlation; blue, negative correlation). The values above/below zero represent positive/negative correlations. Significant correlations are noted by * *p* < 0.05, ** *p* < 0.01.

Cluster-based correlation analysis was performed between shared differential metabolites of the AZM, RH_2_, and COM groups and serum inflammatory cytokine levels or biomarker microbiota. As shown in Figure 9, metabolites including 1-deoxy-D-xylulose 5-phosphate, DL-normetanephrine, indoxyl sulfate, hydrocortisone, phenyllactic acid, and sarcosine showed positive correlations with pro-inflammatory cytokines (IL-1β, IL-6, TNF-α), with hydrocortisone, phenyllactic acid, and sarcosine also negatively correlated with the anti-inflammatory IL-10. These metabolites were positively associated with pathogenic genera such as *Alistipes*, *Mucispirillum*, *Gemella,* and *Escherichia-Shigella*, and negatively correlated with beneficial bacteria, including *Roseburia* and *Lachnospiraceae_FCS020_group*. In contrast, cholesteryl sulfate was negatively correlated with all three pro-inflammatory cytokines and pathogenic genera (*Alistipes* and *Mucispirillum*). Taurochenodeoxycholate showed negative correlations with TNF-α and several pathogens (*Alistipes*, *Gemella*, *Mucispirillum*, and *Ruminococcaceae*), but positive correlations with beneficial taxa such as *Anaerosporomusa*, *Erysipelatoclostridium*, and *Lachnospiraceae_FCS020_group*. Consequently, 1-deoxy-D-xylulose 5-phosphate, DL-normetanephrine, indoxyl sulfate, hydrocortisone, phenyllactic acid, sarcosine, cholesteryl sulfate, and taurochenodeoxycholic acid were identified as key differential metabolites in infectious diarrhea. Their regulation patterns (up/down) were associated with gut microbiota such as *Alistipes*, *Mucispirillum*, and *Roseburia*.

## 4. Discussion

The human gut harbors over a thousand microbial species, forming a complex ecological community [35]. Through interactions with the neural, endocrine, and immune systems, gut microbiota significantly influences host health [36]. Probiotics, defined as live microorganisms that confer a health benefit on the host when administered in adequate amounts [37], improve health by restoring intestinal microbial balance or exerting other functional properties [38].

In this study, a diarrhea model was established by infection with *E. coli* to evaluate the impact of azithromycin on the therapeutic efficacy of *C. butyricum*. The results demonstrated that while azithromycin alleviated diarrheal symptoms in mice, the recovery was unstable and accompanied by shortened colorectal length, persistent inflammatory infiltration, and significant epithelial cell damage and shedding in the mucosal layer. After co-administration with *C. butyricum*, although azithromycin caused intermittent fluctuations in the diarrheal recovery process, the ultimate therapeutic outcomes at the study endpoint were comparable to those of *C. butyricum* monotherapy. These findings indicated that *C. butyricum* can alleviate the side effects caused by long-term use of antibiotics, such as weight loss and shortened colon length, which was consistent with the results reported by Hagihara et al. [39]. Comparative treatment of male and female mice showed that sex-based differences were largely restricted to weight response under *C. butyricum* and combination therapy, with no other significant sex-specific variations observed. In contrast, azithromycin monotherapy caused marked sexual dimorphism in fecal *E. coli* loads and inflammatory cytokine expression, likely due to sex-specific drug metabolism and hormone-mediated immunomodulation. Ultimately, however, the final therapeutic outcome was not sex-dependent.

High-throughput sequencing of the intestinal microbiota of mice showed that diarrhea caused by *E. coli* infection resulted in a decrease in diversity. The MOD group exhibited a distinct microbial profile characterized by significant enrichment of *Alistipes*, *Mucispirillum*, and *Gemella* as indicator genera. *Alistipes* drives disease variability in DSS colitis models by promoting weight loss, intestinal inflammation, and reduced survival [40]. *Mucispirillum* serves as a key indicator of dysbiosis, reflecting a detrimental feedback loop between mucus layer degradation and microbial disruption [41]. *Gemella*, an opportunistic pathogen, exacerbates inflammation through biofilm formation under immune impairment or dysbiosis [42]. In concert with *E. coli*, these genera collectively impair the intestinal barrier integrity and mucosa homeostasis, thereby accelerating inflammatory processes.

The broad-spectrum antibacterial action of azithromycin in the AZM group severely suppressed Gram-positive bacteria, intensifying competition for colonization niches between opportunistic pathogens and antibiotic-resistant strains. *Erysipelatoclostridium* and *Escherichia* are markedly enriched in the gut of inflammatory bowel disease (IBD) patients and serve as marker bacteria for IBD-associated dysbiosis. *Escherichia* species can produce pro-inflammatory polysaccharides and mucin-degrading trans-sialidases that disrupt the intestinal mucosal barrier and exacerbate inflammation [43]. Ning et al. [44] demonstrated that increased gut abundance of *Streptococcus* correlated with elevated systemic inflammatory markers (hs-CRP, neutrophil count, and white blood cell count), suggesting a role in chronic inflammation. Tang et al. further showed that heightened levels of lysophosphatidylcholine, which promotes pro-inflammatory cytokine release and epithelial barrier damage, in colitis mice feces were positively associated with the abundance of *Escherichia* and *Enterorhabdus* [45]. In contrast, while opportunistic pathogens competed for colonization sites, anaerobic gut bacteria such as *Anaerostipes* and *Anaeroplasma* actively counteracted inflammation. *Anaerostipes*, encompassing key butyrate-producing species, synthesizes butyrate by metabolizing sugars, acetate, and lactate, thereby fortifying the intestinal mucosal barrier, modulating immune responses, and suppressing inflammatory pathways [46]. *Anaeroplasma* in turn, exerts anti-inflammatory effects through TGF-β induction, which enhances gut barrier integrity by promoting mucosal IgA production [47].

In the RH_2_ group, *C. butyricum* promoted the colonization of diverse butyrate-producing bacteria. Butyrate enhanced immune regulation by facilitating regulatory T cell (Treg) differentiation, suppressing Th17 cells and pro-inflammatory cytokines (e.g., IL-17, IFN-γ), and activating G protein-coupled receptors to modulate energy metabolism and strengthen intestinal barrier function [48]. Among these bacteria, *Butyricimonas* functioned as a protective agent through its production of butyrate and isobutyrate [49], while *Roseburia* species served as major butyrate producers via the butyryl-CoA: acetate CoA-transferase pathway, aided by efficient dietary fiber utilization [48]. Ruminococcaceae primarily degraded inulin, pectin derivatives, and uronic acids, whereas Lachnospiraceae metabolized starch, arabinoxylan, and inulin—both families generated butyrate through the butyryl-CoA: acetate CoA-transferase pathway [50]. *Muribaculaceae*, which colonize the mucus layer via mucin glycan symbiosis, produced short-chain fatty acids from dietary fiber. Their abundance declined in IBD animal models due to mucosal damage, but rebounded following effective intervention [29]; the elevated *Muribaculaceae* levels in the RH_2_ group thus reflected successful mucosal recovery. Similarly, intervention with *C. butyricum* in the COM group promoted the colonization of *Lachnospiraceae* and *Parabacteroides*. As crucial butyrate producers, *Lachnospiraceae* exert anti-inflammatory effects and nourish colonic epithelial cells by producing butyrate, thereby promoting the expression of tight junction proteins and maintaining the intestinal barrier [51].

**Figure 10:**
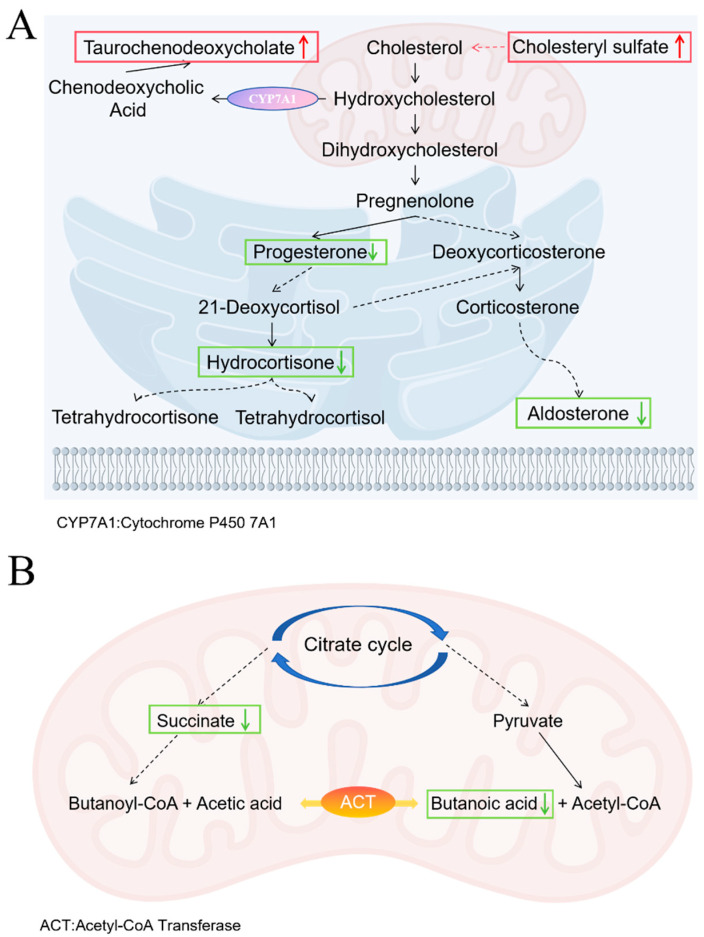
Metabolic Pathway Changes in AZM group. (**A**) Steroid hormone biosynthesis pathway; (**B**) Butyrate metabolic pathways. Upward red arrows indicate upregulation, and downward green arrows indicate downregulation. The dashed arrow indicates a multi-step pathway.

Untargeted metabolomics analysis revealed that after azithromycin treatment, differential metabolites identified in mouse fecal samples were enriched in the steroid hormone biosynthesis and butyrate metabolic pathways. Within the steroid hormone biosynthesis pathway, upstream metabolites (including cholesteryl sulfate and taurochenodeoxycholic acid) showed significant upregulation, while key intermediate and terminal products (such as progesterone, aldosterone, hydrocortisone) were consistently downregulated (Figure 10A). This metabolic profile suggests a potential impairment at a critical step in steroidogenesis. Consequently, the downstream biosynthesis pathways are suppressed. Under intestinal inflammatory conditions, hydroxysteroid sulfotransferase 2B1 (SULT2B1) expression is upregulated, catalyzing cholesterol conversion to cholesteryl sulfate. Elevated cholesteryl sulfate binds to the lysosomal protein NPC2 and competitively inhibits cholesterol transport from lysosomes to the cytoplasm, leading to the endoplasmic reticulum cholesterol levels decereased. This depletion triggers a cellular “cholesterol hunger” signal, which activates the SREBP2-mediated de novo cholesterol synthesis pathway. The accelerated cholesterol production supplies substrates for cell membrane formation, thereby maintaining cellular and tight junction integrity, promoting proliferation, inhibiting apoptosis, and facilitating tissue repair [52]. Meanwhile, cholesterol is converted in the liver by the enzyme CYP7A1 into primary bile acids such as chenodeoxycholic acid. These primary bile acids then conjugate with glycine or taurine to form conjugated bile acids. Under normal conditions, the ileal reabsorption of bile acids is highly efficient; however, when ileal absorption is impaired, they spill into the colon, inhibit water and electrolyte uptake, stimulate secretion, and thereby trigger diarrhea [53]. Thus, although the rise in cholesterol sulfate in the AZM group could promote barrier repair, the disturbed bile-acid metabolism still sustains diarrheal symptoms. In butyrate metabolism, both succinate and butyrate were markedly down-regulated (Figure 10B), because azithromycin broadly suppresses Gram-positive bacteria and thereby weakens the acetyl-CoA transferase-dependent pathway [54]. The resulting butyrate deficiency further compromises barrier function [55], mirroring the metabolic profile observed in ulcerative colitis [56]. These results indicate that, while exerting its antibacterial effect, azithromycin severely disrupts microbial community structure and metabolic homeostasis.

**Figure 11:**
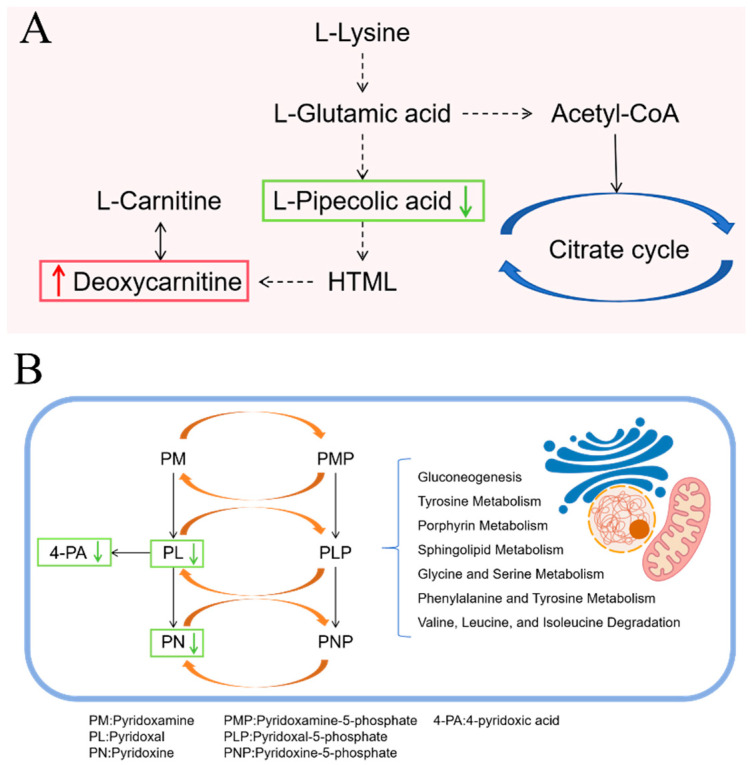
Metabolic Pathway Changes in RH_2_ group. (**A**) Lysine degradation pathway; (**B**) Vitamin B6 metabolic pathways. Upward red arrows indicate upregulation, and downward green arrows indicate downregulation.

In the RH_2_ group treated with *C. butyricum*, differential metabolites from mouse feces were enriched in the lysine degradation and vitamin B6 metabolic pathways. In lysine degradation, upstream L-pipecolic acid was downregulated while deoxycarnitine increased, indicating a shift toward enhanced carnitine synthesis (Figure 11A). Carnitine plays a key role in cellular energy balance and tissue metabolism by promoting fatty acid oxidation to meet elevated energy demands during wound healing. This metabolic shift reduces reliance on glucose, prevents lactate-induced acidification, stabilizes cellular function, and supports energy-intensive processes such as cell proliferation, collagen synthesis, and angiogenesis. Additionally, carnitine downregulates pro-inflammatory factors (e.g., TNF-α, IL-6), promotes anti-inflammatory IL-10 release, and reduces oxidative stress to mitigate tissue damage and accelerate repair [57]. In the vitamin B6 metabolism pathway, levels of pyridoxal, pyridoxine, and 4-pyridoxic acid decreased (Figure 11B). Vitamin B6 serves as an essential cofactor in numerous enzymatic reactions. While vitamin B6 deficiency has been shown to impair lysine degradation and arginine synthesis [58], the well-functioning lysine pathway observed in this study, together with reduced levels of inactive vitamin B6 metabolites, suggests that *C. butyricum* promotes the conversion of inactive forms into active pyridoxal-5′-phosphate. This enhancement likely supports metabolic and functional recovery of damaged intestinal mucosal cells [59].

**Figure 12:**
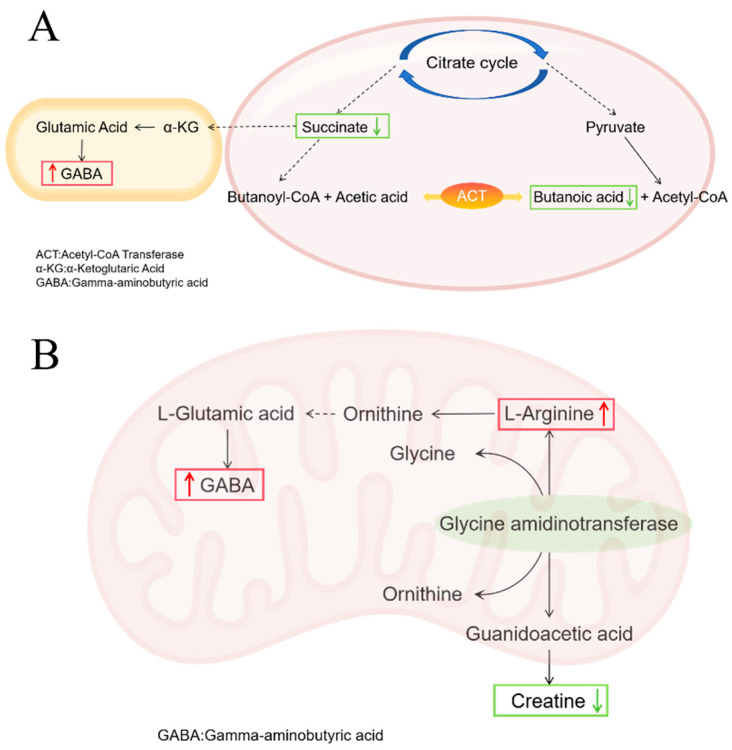
Metabolic Pathway Changes in COM Group. (**A**) Butyric acid metabolic pathway; (**B**) Arginine and proline metabolism pathway. Upward red arrows indicate upregulation, and downward green arrows indicate downregulation.

In the COM group, the relative abundance of gamma-aminobutyric acid (GABA) within the butyrate metabolism pathway was upregulated compared to the MOD group (Figure S4C). This suggests that *C. butyricum* supplementation may have partially shifted this pathway towards activation. Notably, the GABA biosynthesis branch was significantly promoted despite low overall butyrate levels. The concurrent increase in arginine and GABA in the arginine and proline metabolism pathway, alongside a decrease in creatine, points to a potential synergistic support for GABA production (Figure 12B). Given the well-documented role of GABA in reducing intestinal oxidative damage and inflammation [60], its upregulation is a plausible contributor to the suppression of pro-inflammatory cytokines (e.g., IL-1β, IL-6). Furthermore, GABA’s ability to enhance tight junction protein expression and alleviate oxidative stress probably underlies the improved intestinal barrier integrity observed in our study. Arginine, as a precursor for nitric oxide and polyamines, plays a pivotal role in enhancing mucosal blood flow, promoting epithelial cell proliferation, and facilitating tissue repair [61]. Its immunomodulatory functions likely contributed to the resolution of inflammation [62,63]. The co-upregulation of arginine and GABA suggests a coordinated metabolic response: while GABA provides anti-inflammatory and neuromodulatory benefits, arginine drives the structural regeneration of the damaged intestinal epithelium. This synergistic action effectively addresses both the inflammatory response and the physical barrier damage caused by the infection. The vitamin B6 metabolic pathway in the COM group remained consistent with that observed in the RH_2_ group.

Furthermore, correlation analysis indicated that two differential metabolites, 1-deoxy-D-xylulose 5-phosphate and indoxyl sulfate, which play key roles in infectious diarrhea, are derived from pathogenic bacterial metabolism. Their elevated levels were directly linked to increased abundance of *Alistipes* and *Mucispirillum*. 1-deoxy-D-xylulose 5-phosphate is a metabolic intermediate synthesized by pathogens such as *E. coli* and *Mycobacterium tuberculosis* via the methylerythritol phosphate pathway. By supporting pathogen proliferation, it indirectly compromises intestinal health and exacerbates infection [64]. Indoxyl sulfate production relies on the microbial metabolism of tryptophan by pathogens including *E. coli*. The expansion of these toxin-producing bacteria leads to accumulation of uremic toxins, resulting in tissue injury through pro-inflammatory and pro-oxidative mechanisms [65]. Prolonged elevation of hydrocortisone may heighten infection risk by suppressing Th1 immune responses and impairing gut health [66]. In contrast, *Roseburia* has been shown to effectively attenuate abnormal hydrocortisone increases.

In conclusion, this study demonstrates that *C. butyricum* exhibits a strong therapeutic effect in a mouse model of *E. coli*-induced infectious diarrhea. While azithromycin effectively suppressed pathogenic *E. coli*, its use was associated with significant disruptions to intestinal microbiota composition and metabolic homeostasis, potentially delaying symptomatic and histological recovery. Notably, when administered in combination with azithromycin, *C. butyricum* retained considerable efficacy, ameliorating dysbiosis and promoting recovery, though its regulatory impact on the gut homeostasis appeared partially attenuated compared to monotherapy. These findings suggest that *C. butyricum* can remain functionally active even during antibiotic exposure. Future studies should focus on directly quantifying *C. butyricum* colonization dynamics using strain-specific approaches to validate its survival and activity in vivo. These results support the potential clinical value of supplementing with *C. butyricum* during antibiotic treatment to mitigate microbiota-related side effects. Further investigation is needed to determine whether continuing probiotic administration after antibiotic cessation could enhance long-term microbial stability and clinical outcomes.

## Figures and Tables

**Table 1:** Behavioral Scoring Criteria.

Category	Scoring Criteria	Score
Coat Condition	Normal, smooth fur	0
	Mildly unkempt fur	1
	Pilorection with loss of gloss	2
	Severe pilorection with visible hair loss	3
	All the above signs with half-closed eyes	4
Spontaneous Behavior	active, normal exploration	0
	Slightly reduced activity	1
	Minimal movement, huddling	2
	Marked restlessness or lethargy	3
Response to Stimulation	Alert and reactive	0
	Sluggish or unresponsive	3

**Table 2:** Fecal Status Score.

Score	Fecal Characteristics
0	normal feces
1	loose feces
2	Semi-formed feces
3	degree of diarrhea
4	Watery diarrhea or difficulty in defecation

## Data Availability

The original contributions presented in this study are included in the article/Supplementary Materials. Further inquiries can be directed to the corresponding author.

## Supplementary Materials

The following supporting information can be downloaded at: https://www.mdpi.com/article/10.3390/microorganisms13122812/s1, Figure S1: Scoring of Behavioral States; Figure S2: Fecal Status Scoring of Mice; Figure S3: LEfSe and STAMP analysis results; Figure S4: Heatmap of Differential Metabolite Abundance in Mouse Feces; Figure S5: Differential Metabolite Venn Diagram; Figure S6: Relative abundance of shared differential metabolites and Butanoic acid; Figure S7: Pathway enrichment analysis.

## References

[1] Ramirez: J., Guarner F., Bustos Fernandez L., Maruy A., Sdepanian V.L., Cohen H. (2020). Antibiotics as major disruptors of gut microbiota. Front. Cell. Infect. Microbiol..

[2] Huang C., Feng S., Huo F., Liu H. (2022). Effects of four antibiotics on the diversity of the intestinal microbiota. Microbiol. Spectrum.

[3] Fishbein S.R., Mahmud B., Dantas G. (2023). Antibiotic perturbations to the gut microbiome. Nat. Rev. Microbiol..

[4] Li W., Zhang S., Wang Y., Bian H., Yu S., Huang L., Ma W. (2023). Complex probiotics alleviate ampicillin-induced antibiotic-associated diarrhea in mice. Front. Microbiol..

[5] Shin E., Paek J.J., Lee Y. (2023). Antimicrobial resistance of seventy lactic acid bacteria isolated from commercial probiotics in Korea. J. Microbiol. Biotechnol..

[6] Gu S.-L., Gong Y., Zhang J., Chen Y., Wu Z., Xu Q., Fang Y., Wang J., Tang L.-L. (2020). Effect of the short-term use of fluoroquinolone and β-lactam antibiotics on mouse gut microbiota. Infect. Drug Resist..

[7] Ianiro G., Tilg H., Gasbarrini A.J.G. (2016). Antibiotics as deep modulators of gut microbiota: Between good and evil. Gut.

[8] Pei Z., Liu Y., Yi Z., Liao J., Wang H., Zhang H., Chen W., Lu W. (2023). Diversity within the species *Clostridium butyricum*: Pan-genome, phylogeny, prophage, carbohydrate utilization, and antibiotic resistance. J. Appl. Microbiol..

[9] Yang Y., Shao Y., Pei C., Liu Y., Zhang M., Zhu X., Li J., Feng L., Li G., Li K. (2024). Pangenome analyses of *Clostridium butyricum* provide insights into its genetic characteristics and industrial application. Genomics.

[10] Franciosa G., Scalfaro C., Di Bonito P., Vitale M., Aureli P. (2011). Identification of novel linear megaplasmids carrying a ß-lactamase gene in neurotoxigenic *Clostridium butyricum* type E strains. PLoS ONE.

[11] Yang Y.-M., Zhang M.-Y., Wu Y.-Y., Zhang L., Zhang Y.-X. (2025). Survival and Morphological Changes of *Clostridium butyricum* Spores Co-Exposed to Antibiotics and Simulated Gastrointestinal Fluids: Implications for Antibiotic Stewardship. Microorganisms.

[12] Hagihara M., Yamashita R., Matsumoto A., Mori T., Kuroki Y., Kudo H., Oka K., Takahashi M., Nonogaki T., Yamagishi Y. (2018). The impact of *Clostridium butyricum* MIYAIRI 588 on the murine gut microbiome and colonic tissue. Anaerobe.

[13] Hagihara M., Yamashita R., Matsumoto A., Mori T., Inagaki T., Nonogaki T., Kuroki Y., Higashi S., Oka K., Takahashi M. (2019). The impact of probiotic *Clostridium butyricum* MIYAIRI 588 on murine gut metabolic alterations. J. Infect. Chemother..

[14] Peterson E., Kaur P. (2018). Antibiotic resistance mechanisms in bacteria: Relationships between resistance determinants of antibiotic producers, environmental bacteria, and clinical pathogens. Front. Microbiol..

[15] Zhao X., Yang J., Ju Z., Wu J., Wang L., Lin H., Sun S. (2020). *Clostridium butyricum* ameliorates Salmonella enteritis induced inflammation by enhancing and improving immunity of the intestinal epithelial barrier at the intestinal mucosal level. Front. Microbiol..

[16] Zhang X., Song M., Lv P., Hao G., Sun S. (2022). Effects of *Clostridium butyricum* on intestinal environment and gut microbiome under Salmonella infection. Poult. Sci..

[17] Huang T., Peng X.-Y., Gao B., Wei Q.-L., Xiang R., Yuan M.-G., Xu Z.-H. (2019). The effect of *Clostridium butyricum* on gut microbiota, immune response and intestinal barrier function during the development of necrotic enteritis in chickens. Front. Microbiol..

[18] Li W., Xu B., Wang L., Sun Q., Deng W., Wei F., Ma H., Fu C., Wang G., Li S. (2021). Effects of *Clostridium butyricum* on growth performance, gut microbiota and intestinal barrier function of broilers. Front. Microbiol..

[19] Wu J., Wang J., Lin Z., Liu C., Zhang Y., Zhang S., Zhou M., Zhao J., Liu H., Ma X. (2023). *Clostridium butyricum* alleviates weaned stress of piglets by improving intestinal immune function and gut microbiota. Food Chem..

[20] Li Y., Liu M., Liu H., Sui X., Liu Y., Wei X., Liu C., Cheng Y., Ye W., Gao B. (2021). The anti-inflammatory effect and mucosal barrier protection of *Clostridium butyricum* RH2 in ceftriaxone-induced intestinal dysbacteriosis. Front. Cell. Infect. Microbiol..

[21] Li Z., He H., Chen M., Ni M., Guo C., Wan Z., Zhou J., Wang Z., Wang Y., Cai H. (2024). Novel mechanism of *Clostridium butyricum* alleviated coprophagy prevention-induced intestinal inflammation in rabbit. Int. Immunopharmacol..

[22] Guo Z., Qian Y., Peng X., Qin C., Ren H., Du J., Huang C., Pan M., Ou W. (2025). Effects of Dietary *Clostridium butyricum* on Growth and Intestinal Mucosal Barrier Functions of Juvenile Channel Catfish (*Ictalurus punctatus*). Microorganisms.

[23] Zhao J., Jiang L., He W., Han D., Yang X., Wu L., Zhong H. (2024). *Clostridium butyricum*, a future star in sepsis treatment. Front. Cell. Infect. Microbiol..

[24] Yu S., Xie J., Guo Q., Yan X., Wang Y., Leng T., Li L., Zhou J., Zhang W., Su X. (2024). *Clostridium butyricum* isolated from giant panda can attenuate dextran sodium sulfate-induced colitis in mice. Front. Microbiol..

[25] Xu J., Xu H., Li J., Huang W., Li Y., Guo X., Zhu M., Peng Y., Zhou Y., Nie Y. (2025). *Clostridium butyricum*-induced balance in colonic retinol metabolism and short-chain fatty acid levels inhibit IgA-related mucosal immunity and relieve colitis developments. Microbiol. Res..

[26] Ma L., Shen Q., Lyu W., Lv L., Wang W., Yu M., Yang H., Tao S., Xiao Y. (2022). *Clostridium butyricum* and its derived extracellular vesicles modulate gut homeostasis and ameliorate acute experimental colitis. Microbiol. Spectrum.

[27] Hua D., Yang Q., Li X., Zhou X., Kang Y., Zhao Y., Wu D., Zhang Z., Li B., Wang X. (2025). The combination of *Clostridium butyricum* and *Akkermansia muciniphila* mitigates DSS-induced colitis and attenuates colitis-associated tumorigenesis by modulating gut microbiota and reducing CD8+ T cells in mice. mSystems.

[28] Mühlen S., Ramming I., Pils M.C., Koeppel M., Glaser J., Leong J., Flieger A., Stecher B., Dersch P. (2020). Identification of antibiotics that diminish disease in a murine model of enterohemorrhagic *Escherichia coli* infection. Antimicrob. Agents Chemother..

[29] Zhu Y., Chen B., Zhang X., Akbar M.T., Wu T., Zhang Y., Zhi L., Shen Q. (2024). Exploration of the muribaculaceae family in the gut microbiota: Diversity, metabolism, and function. Nutrients.

[30] Chang K.C., Nagarajan N., Gan Y.-H. (2024). Short-chain fatty acids of various lengths differentially inhibit Klebsiella pneumoniae and Enterobacteriaceae species. mSphere.

[31] Zhang M., Cui Y., Liu P., Mo R., Wang H., Li Y., Wu Y. (2024). Oat β-(1 → 3, 1 → 4)-d-glucan alleviates food allergy-induced colonic injury in mice by increasing Lachnospiraceae abundance and butyrate production. Carbohydr. Polym..

[32] Benítez-Páez A., Gomez del Pugar E.M., López-Almela I., Moya-Pérez Á., Codoñer-Franch P., Sanz Y. (2020). Depletion of Blautia species in the microbiota of obese children relates to intestinal inflammation and metabolic phenotype worsening. mSystems.

[33] Cui Y., Zhang L., Wang X., Yi Y., Shan Y., Liu B., Zhou Y., Lü X. (2022). Roles of intestinal Parabacteroides in human health and diseases. FEMS Microbiol. Lett..

[34] Zhou H., Huang D., Sun Z., Chen X. (2024). Effects of intestinal Desulfovibrio bacteria on host health and its potential regulatory strategies: A review. Microbiol. Res..

[35] Lynch S.V., Pedersen O. (2016). The human intestinal microbiome in health and disease. N. Engl. J. Med..

[36] Cryan J.F., O’Riordan K.J., Cowan C.S., Sandhu K.V., Bastiaanssen T.F., Boehme M., Codagnone M.G., Cussotto S., Fulling C., Golubeva A.V. (2019). The microbiota-gut-brain axis. Physiol. Rev..

[37] Hill C., Guarner F., Reid G., Gibson G.R., Merenstein D.J., Pot B., Morelli L., Canani R.B., Flint H.J., Salminen S. (2014). The International Scientific Association for Probiotics and Prebiotics consensus statement on the scope and appropriate use of the term probiotic. Nat. Rev. Gastroenterol. Hepatol..

[38] Szajewska H., Scott K.P., de Meij T., Forslund-Startceva S.K., Knight R., Koren O., Little P., Johnston B.C., Łukasik J., Suez J. (2024). Antibiotic-perturbed microbiota and the role of probiotics. Nat. Rev. Gastroenterol. Hepatol..

[39] Hagihara M., Kuroki Y., Ariyoshi T., Higashi S., Fukuda K., Yamashita R., Matsumoto A., Mori T., Mimura K., Yamaguchi N. (2020). *Clostridium butyricum* modulates the microbiome to protect intestinal barrier function in mice with antibiotic-induced dysbiosis. iScience.

[40] Forster S.C., Clare S., Beresford-Jones B.S., Harcourt K., Notley G., Stares M.D., Kumar N., Soderholm A.T., Adoum A., Wong H. (2022). Identification of gut microbial species linked with disease variability in a widely used mouse model of colitis. Nat. Microbiol..

[41] Berry D., Schwab C., Milinovich G., Reichert J., Ben Mahfoudh K., Decker T., Engel M., Hai B., Hainzl E., Heider S. (2012). Phylotype-level 16S rRNA analysis reveals new bacterial indicators of health state in acute murine colitis. ISME J..

[42] García López E., Martín-Galiano A.J. (2020). The versatility of opportunistic infections caused by Gemella isolates is supported by the carriage of virulence factors from multiple origins. Front. Microbiol..

[43] Shawki A., McCole D.F. (2017). Mechanisms of intestinal epithelial barrier dysfunction by adherent-invasive *Escherichia coli*. Cell. Mol. Gastroenterol. Hepatol..

[44] Ning L., Zhou Y.-L., Sun H., Zhang Y., Shen C., Wang Z., Xuan B., Zhao Y., Ma Y., Yan Y. (2023). Microbiome and metabolome features in inflammatory bowel disease via multi-omics integration analyses across cohorts. Nat. Commun..

[45] Tang X., Wang W., Hong G., Duan C., Zhu S., Tian Y., Han C., Qian W., Lin R., Hou X. (2021). Gut microbiota-mediated lysophosphatidylcholine generation promotes colitis in intestinal epithelium-specific Fut2 deficiency. J. Biomed. Sci..

[46] Liu D., Xie L.-S., Lian S., Li K., Yang Y., Wang W.-Z., Hu S., Liu S.-J., Liu C., He Z. (2024). *Anaerostipes hadrus*, a butyrate-producing bacterium capable of metabolizing 5-fluorouracil. mSphere.

[47] Xu X., Davelaar N., Otten-Mus A., Asmawidjaja P., Hazes J., Baeten D., Vis M., Bisoendial R., Lubberts E. (2019). P103/O14 Interleukin-17 is produced by CD4+ but not CD8+ T cells after TCR activation in synovial fluid of psoriatic arthritis patients. Ann. Rheum. Dis..

[48] Nie K., Ma K., Luo W., Shen Z., Yang Z., Xiao M., Tong T., Yang Y., Wang X. (2021). Roseburia intestinalis: A beneficial gut organism from the discoveries in genus and species. Front. Cell. Infect. Microbiol..

[49] Liang X., Fu Y., Cao W.-T., Wang Z., Zhang K., Jiang Z., Jia X., Liu C.-Y., Lin H.-R., Zhong H. (2022). Gut microbiome, cognitive function and brain structure: A multi-omics integration analysis. Transl. Neurodegener..

[50] Louis P., Flint H.J. (2017). Formation of propionate and butyrate by the human colonic microbiota. Environ. Microbiol..

[51] Duan J., Li Q., Cheng Y., Zhu W., Liu H., Li F. (2024). Therapeutic potential of Parabacteroides distasonis in gastrointestinal and hepatic disease. MedComm.

[52] Xu D., Ma R., Ju Y., Song X., Niu B., Hong W., Wang R., Yang Q., Zhao Z., Zhang Y. (2022). Cholesterol sulfate alleviates ulcerative colitis by promoting cholesterol biosynthesis in colonic epithelial cells. Nat. Commun..

[53] Rao A.S., Wong B.S., Camilleri M., Odunsi–Shiyanbade S.T., McKinzie S., Ryks M., Burton D., Carlson P., Lamsam J., Singh R. (2010). Chenodeoxycholate in females with irritable bowel syndrome-constipation: A pharmacodynamic and pharmacogenetic analysis. Gastroenterology.

[54] Hays K.E., Pfaffinger J.M., Ryznar R. (2024). The interplay between gut microbiota, short-chain fatty acids, and implications for host health and disease. Gut Microbes.

[55] Deng B., Liu Y., Chen Y., He P., Ma J., Tan Z., Zhang J., Dong W. (2024). Exploring the butyrate metabolism-related shared genes in metabolic associated steatohepatitis and ulcerative colitis. Sci. Rep..

[56] Franzosa E.A., Sirota-Madi A., Avila-Pacheco J., Fornelos N., Haiser H.J., Reinker S., Vatanen T., Hall A.B., Mallick H., McIver L.J. (2019). Gut microbiome structure and metabolic activity in inflammatory bowel disease. Nat. Microbiol..

[57] Flanagan J.L., Simmons P.A., Vehige J., Willcox M.D., Garrett Q. (2010). Role of carnitine in disease. Nutr. Metab..

[58] Mayengbam S., Chleilat F., Reimer R.A. (2020). Dietary vitamin B6 deficiency impairs gut microbiota and host and microbial metabolites in rats. Biomedicines.

[59] Ueland P.M., McCann A., Midttun Ø., Ulvik A. (2017). Inflammation, vitamin B6 and related pathways. Mol. Asp. Med..

[60] Deng Z., Li D., Wang L., Lan J., Wang J., Ma Y. (2024). Activation of GABABR attenuates intestinal inflammation by reducing oxidative stress through modulating the TLR4/MyD88/NLRP3 pathway and gut microbiota abundance. Antioxidants.

[61] Kurhaluk N., Tkaczenko H. (2025). L-Arginine and Nitric Oxide in Vascular Regulation—Experimental Findings in the Context of Blood Donation. Nutrients.

[62] Zhang B., Li G., Shahid M.S., Gan L., Fan H., Lv Z., Yan S., Guo Y. (2020). Dietary l-arginine supplementation ameliorates inflammatory response and alters gut microbiota composition in broiler chickens infected with Salmonella enterica serovar Typhimurium. Poult. Sci..

[63] Zhang H., Peng A., Yu Y., Guo S., Wang M., Wang H. (2019). L-arginine protects ovine intestinal epithelial cells from lipopolysaccharide-induced apoptosis through alleviating oxidative stress. J. Agric. Food. Chem..

[64] Gierse R.M., Oerlemans R., Reddem E.R., Gawriljuk V.O., Alhayek A., Baitinger D., Jakobi H., Laber B., Lange G., Hirsch A.K. (2022). First crystal structures of 1-deoxy-D-xylulose 5-phosphate synthase (DXPS) from Mycobacterium tuberculosis indicate a distinct mechanism of intermediate stabilization. Sci. Rep..

[65] Hung S.C., Kuo K.L., Wu C.C., Tarng D.C. (2017). Indoxyl sulfate: A novel cardiovascular risk factor in chronic kidney disease. J. Am. Heart Assoc..

[66] Duran-Pinedo A.E., Solbiati J., Frias-Lopez J. (2018). The effect of the stress hormone cortisol on the metatranscriptome of the oral microbiome. npj Biofilms Microbiomes.

