# Supplementary material for: Combination Therapy with Azithromycin and Clostridium butyricum Retains Anti-Diarrheal Efficacy but Partially Compromises Gut Microbiota Restoration Compared to Probiotics Monotherapy
Source: Microorganisms. 2025 Dec 10;13(12):2812. doi: 10.3390/microorganisms13122812 (PMC12736042; doi:10.3390/microorganisms13122812)
Supplement: Supplementary file 1 [file microorganisms-13-02812-s001.zip › microorganisms-4006398-supplementary.pdf]

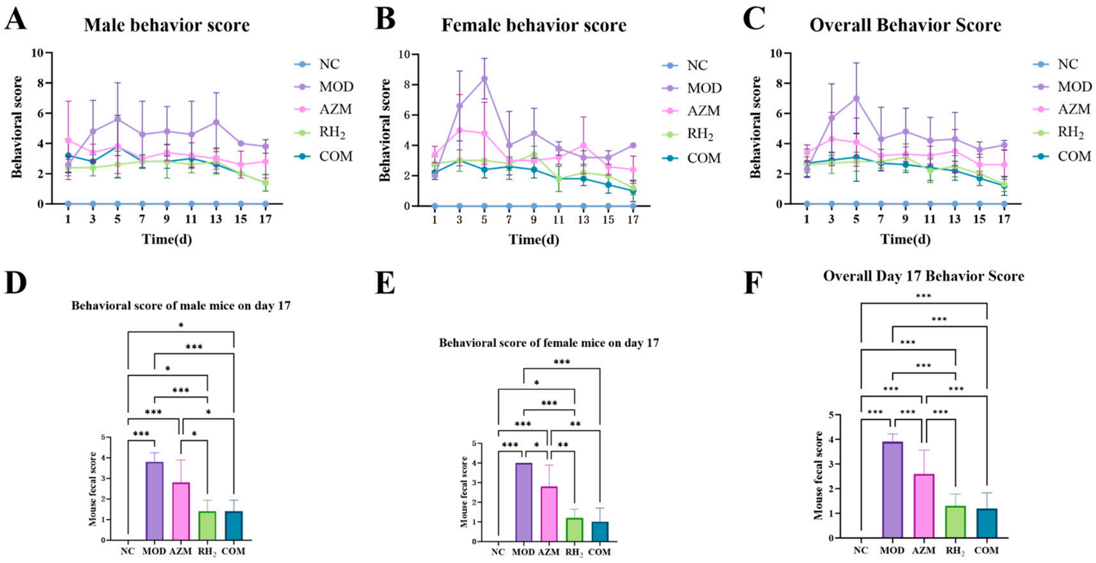

Figure S1 Scoring of Behavioral States. (A) Male mice; (B) Female mice; (C) Overall mice; (D) Male mice (day 17); (E) Female mice (day 17); (F) Overall mice (day 17) (*$p < 0.05$, **$p < 0.01$, ***$p < 0.001$, Male and Female n = 5, Overall n = 10)

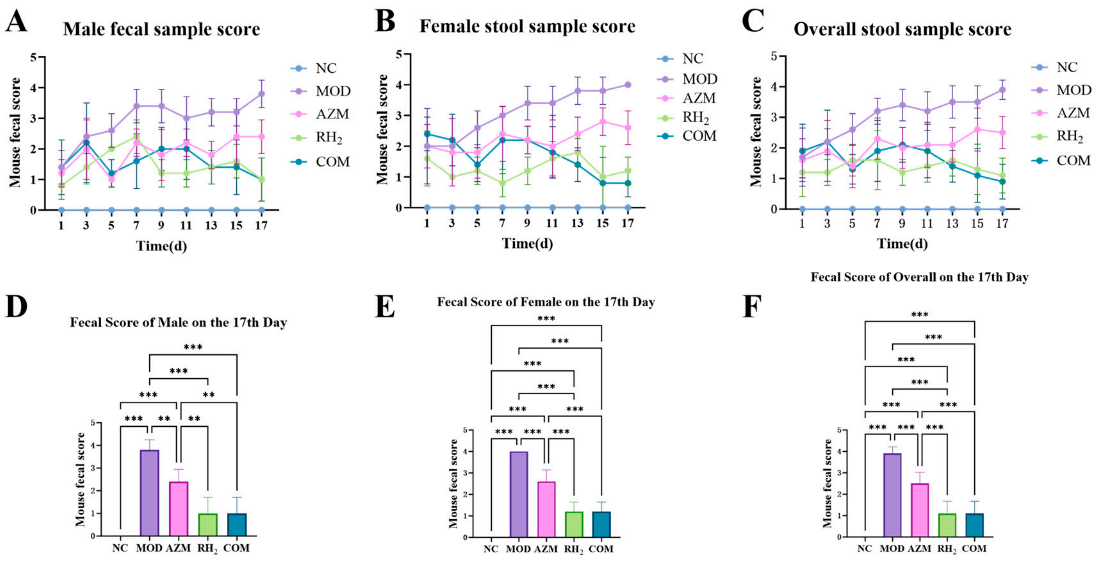

Figure S2 Fecal Status Scoring of Mice. (A) Male; (B) Female; (C) Overall; (D) Male (Day 17); (E) Female (Day 17) (**$p < 0.01$, ***$p < 0.001$, Male and Female n = 5, Overall n = 10)

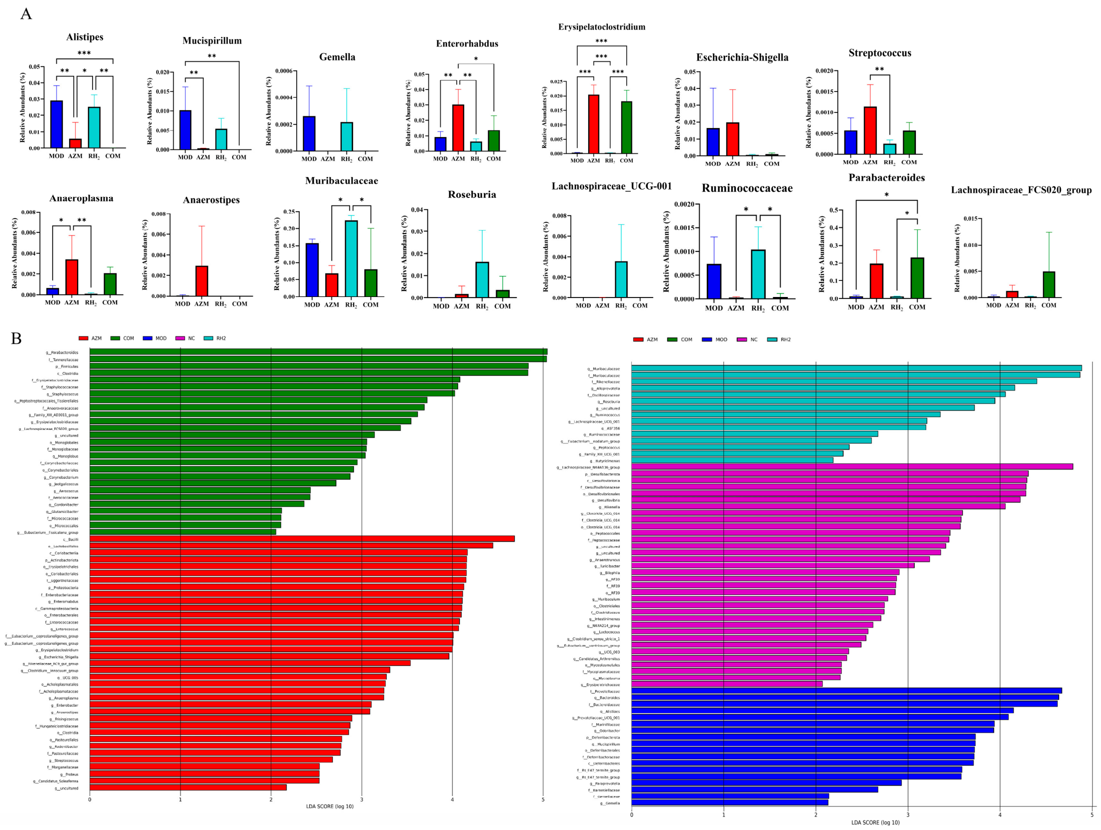

Figure S3 LEfSe and STAMP analysis results. (A) LDA Score Distribution; (B) Biomarker Taxa

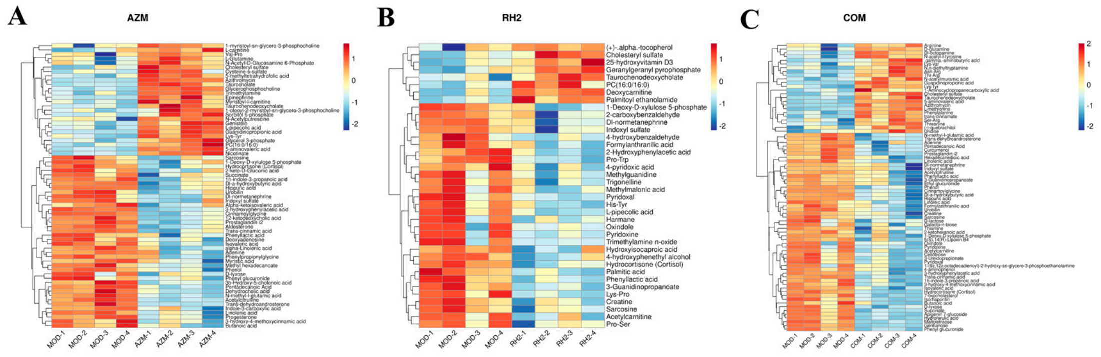

Figure S4 Heatmap of Differential Metabolite Abundance in Mouse Feces. (A) AZM; (B) RH_2; (C) COM

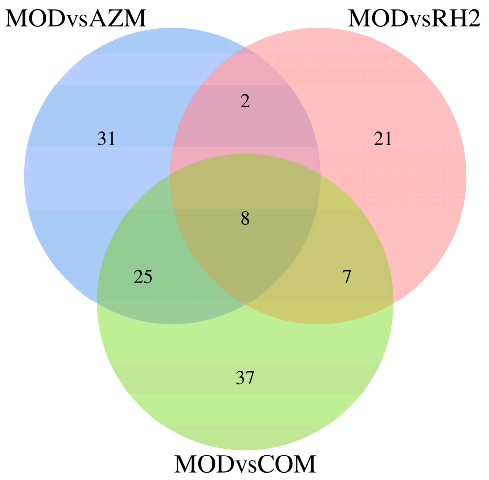

Figure S5 Differential Metabolite Venn Diagram

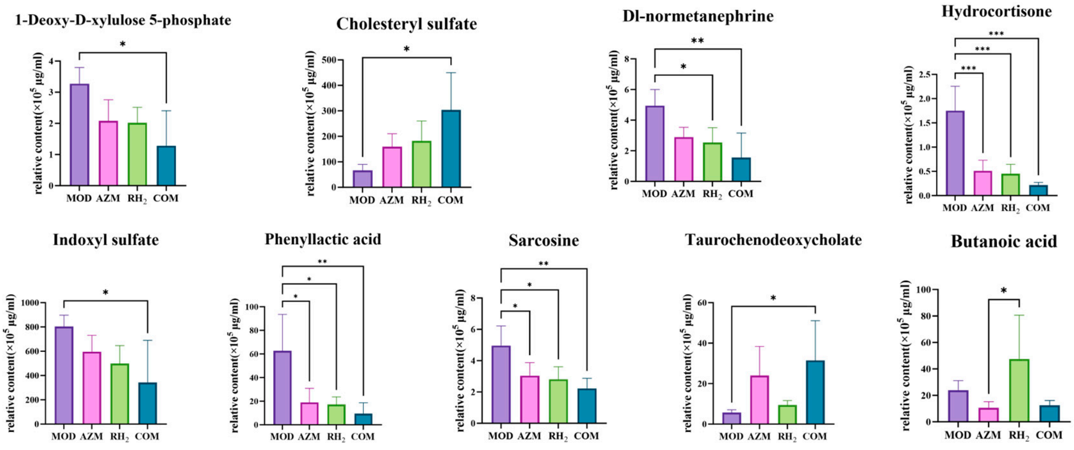

Figure S6 Relative abundance of shared differential metabolites and Butanoic acid

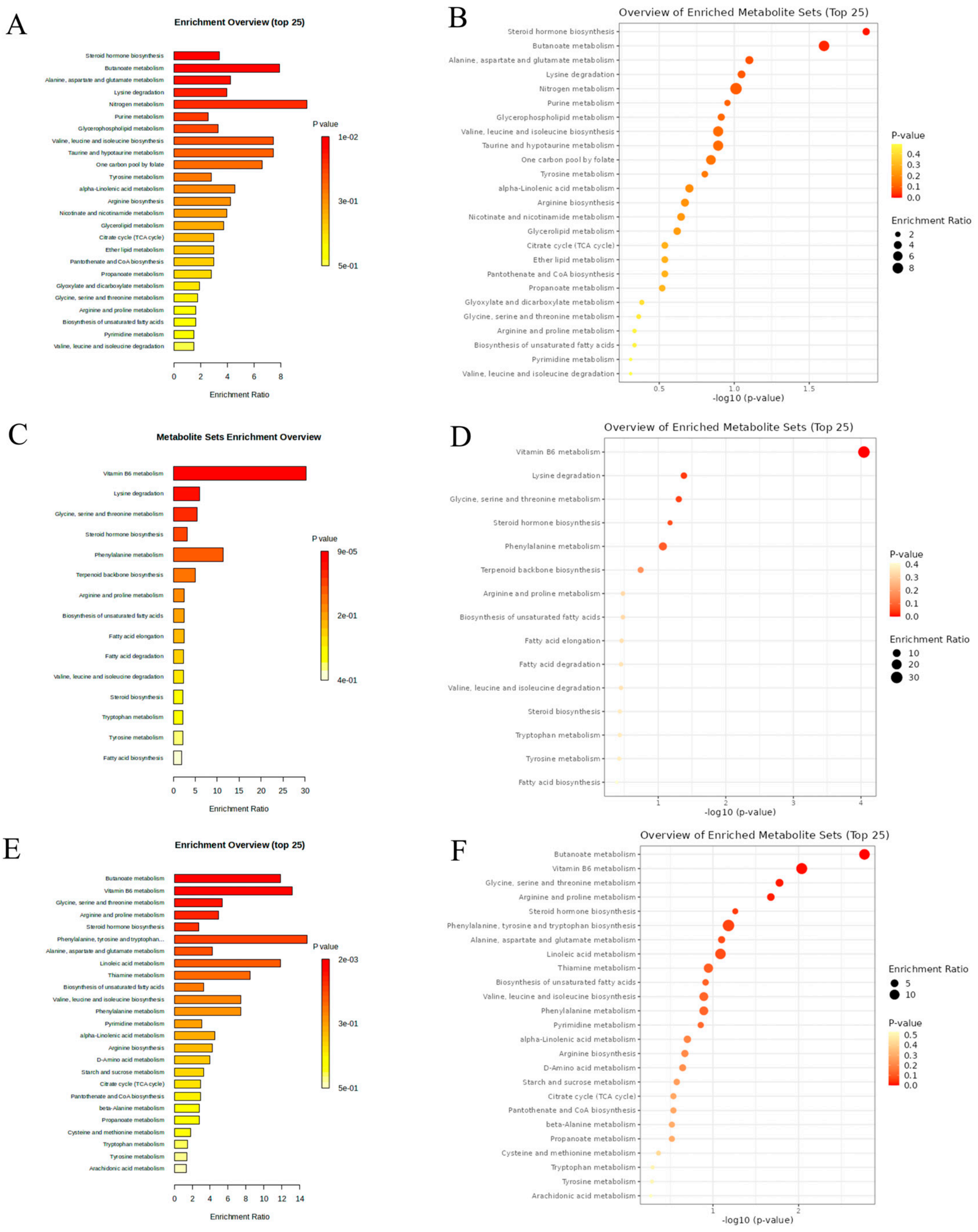

Figure S7 Pathway enrichment analysis. (A & B) AZM; (C & D) RH_2; (E & F) COM
